# Prenatal cannabinoid exposure: why expecting individuals should take a pregnancy pause from using cannabinoid products

**DOI:** 10.3389/fped.2023.1278227

**Published:** 2023-10-11

**Authors:** Alexis Lin, Gelonia L. Dent, Suzy Davies, Zarena M. Dominguez, Leigh-Anne Cioffredi, Gabrielle L. McLemore, Jessie R. Maxwell

**Affiliations:** ^1^ Gustavus Adolphus College, Saint Peter, MN, United States; ^2^ Department of Mathematics, Medgar Evers College, CUNY, Brooklyn, NY, United States; ^3^ Department of Neurosciences, University of New Mexico, Albuquerque, NM, United States; ^4^ Department of Pediatrics, University of New Mexico, Albuquerque, NM, United States; ^5^ Department of Pediatrics, University of Vermont, Burlington, VT, United States; ^6^ Department of Biology, Morgan State University, Baltimore, MD, United States

**Keywords:** prenatal cannabinoid exposure, prenatal cannabis exposure, neurodevelopmental outcome, intrauterine exposure, prenatal marijuana exposure

## Abstract

Cannabinoid use in all populations is increasing as legalization across the United States continues. Concerningly, there is a lack of caution provided by medical providers to pregnant individuals as to the impact the use of cannabinoids could have on the developing fetus. Research continues in both the preclinical and clinical areas, and is severely needed, as the potency of delta-9-tetrahydrocannabinol (THC), the primary psychoactive component of cannabis, has increased dramatically since the initial studies were completed. Thus far, clinical studies raise compelling evidence for short term memory deficits, impulse control issues, and attention deficiencies following prenatal cannabinoid exposure (PCE). These changes may be mediated through epigenetic modifications that not only impact the current offspring but could carry forward to future generations. While additional studies are needed, a pregnancy pause from cannabinoid products should be strongly recommended by providers to ensure the optimal health and well-being of our future generations.

## Introduction

### Cannabinoids and the extended endocannabinoid system (Endocannabinoidome)

The chemical nature of the *Cannabis sativa* plant (i.e., cannabis or marijuana) is complicated due to the presence of over 500 unique chemical compounds, more than 100 of which are a class of lipophilic chemicals known as phytocannabinoids or cannabinoids (CB) (1–3). Natural CB includes delta-9-tetrahydrocannabinol (THC), the primary psychoactive phytoCB (4), and cannabidiol (5) (CBD), the main non-psychoactive phytoCB in marijuana. Cannabinoids act on the G protein-coupled CB receptors, CB1R (6) and CB2R (7). Additionally, the endocannabinoid system (eCB) consists of the endogenous cannabinoids: N-arachidonoylethanolamine (anandamide; AEA) and 2-arachidonoylglycerol (2-AG), and their biosynthetic enzymes, *N*-acyl-phosphatidylethanolamine-hydrolyzing phospholipase D (NAPE-PLD) and sn-1-diacylglycerol lipases (DAGL), and their degradative enzymes, fatty acid amide hydrolase 1 (FAAH) and monoacylglycerol lipase (MGL) (8, 9). From 1970 to 2019, for both herbal cannabis and cannabis resin, the mean concentrations of THC increased. For this same period, there was no such increase in CBD concentration in herbal cannabis or cannabis resin (10). Between 2018 and 2019, the THC:CBD mean ratios (∼54:1 and ∼25:1, respectively) decreased by about 54%, indicating a preference for recreational cannabis use products containing a high-THC to low-CBD ratio (3). The near-ubiquitous neuromodulatory nature of the eCB is attributable to its homeostatic role in the modulation of neurodevelopment, neuroplasticity, several physiological and cognitive processes, and its responses to endogenous and exogenous perturbations (11–13). The homeostatic function of the eCB is to maintain the internal milieu (e.g., immunoregulation, mood regulation, and thermoregulation) and bioenergetic balance (i.e., energy flow through biological systems).

Moreover, the eCB effects nervous system development and function, fertility, pregnancy, and perinatal development. In addition, several components of the eCB have been identified in the embryo, follicular fluid, ovaries, the placenta, and the uterus (14, 15). The various constituents of the eCB system have both pro-homeostatic roles in the maintenance of health and well-being, and anti-homeostatic roles in the genesis of several diseases such as neurodegenerative diseases, cancer, and cardiovascular disease. Therefore, it is feasible that the eCB is a prime target in developing novel cannabimimetic therapeutic agents capable of modulating this system via inhibition or disinhibition of metabolic pathways or CB receptor agonism or antagonism (16). THC is now the most used psychoactive substance in the United States, with utilization continuing to rise as Americans support the legalization of recreational use nationwide (17). Concurrently, most adults perceive that THC is harmless during pregnancy (18, 19). Thus, it is imperative that additional research is conducted to determine the risks of THC use during pregnancy.

## Prevalence of cannabinoid use

### Has the prevalence of cannabinoid use increased with the legalization of cannabinoids for medicinal purposes?

Cannabinoids are the most widely used, federally illegal, recreational drug in the United States. The increased prevalence of cannabis use is partly due to its ever-increasing potency, availability, social acceptance, perceived safety, and access due to extensive legalization of cannabinoids for medical and recreational use in many states and worldwide (20, 21). Coinciding with the increased prevalence of cannabis containing higher concentrations of THC, the pattern of cannabinoid use is evolving towards almost daily use as compared to previous use patterns (22). While 55 million Americans report use of cannabis in the last year, which now surpasses the number of tobacco smokers, 1 in 16 high school seniors report daily use of cannabis products (23).

There exist several routes of exposure for cannabis including smoking the flowers and leaves, vaping concentrates, spraying a vaporized solution on the buccal mucosa, ingesting capsules, liquids, or foods, and applying topical lotions (24). The prevalence of cannabis vaping among adolescents has increased significantly, suggesting that the preference for cannabis products may be changing from smoking the dried herb to vaping cannabis oil (25, 26). Monitoring the Future is an ongoing survey funded by the National Institutes of Health that has measured drug and alcohol use since 1975. From 2017 to 2018, young adults were observed to double their past 30-day cannabis vaping use, which was among the largest 1-year increase in any substance use recorded in Monitoring the Future (27). Unfortunately, the following year showed a doubling of use for youth (27). A 2017 North Carolina Youth Tobacco Survey revealed that 1 in 10 high school students reported vaping cannabis (28). Recently, the FDA has made the public aware of reports numbering in the hundreds of vaping-associated severe lung illnesses and several deaths (29).

With the increased prevalence of vaping, there has been a corresponding increase in the vaping of new synthetic designer CB. Neocannabinoids (nCB) are the newest iteration of CB-like drugs that are intended to mimic THC but are chemical concoctions of various non-CB psychoactive stimulants that are not canonical CB receptor ligands. These chemicals can be sprayed on marijuana leaves and smoked to achieve a desired state of euphoria and are sold under a variety of names/street names that tout special effects (e.g., Black Magic, Crazy Clown, Paradise, Serenity, Spice). From 2009 to 2020, law enforcement agencies have witnessed a proliferation of hundreds of different designer nCB that are hyped and marketed as “legal” substitutes for marijuana and sold under the guise of “herbal incense” or “potpourri” (30, 31). Initially, these synthetic drugs were designed as pharmacological tools to interrogate the eCB and were anticipated to be innovative pharmacotherapeutic agents, now, these once promising nCB are highly abused for their extremely potent psychoactive properties (11, 32–34) With the proliferation of these new and potentially dangerous synthetic drugs flooding the market and the increasing quest of the user to obtain a greater “high,” there exists the distinct possibility that clinicians will soon witness an explosion in the prevalence of perinatal cannabinoid use.

## Prevalence of perinatal cannabinoid Use

### Has the proliferation of nCB contributed to the increased prevalence of perinatal cannabinoid use?

**Figure 1:**
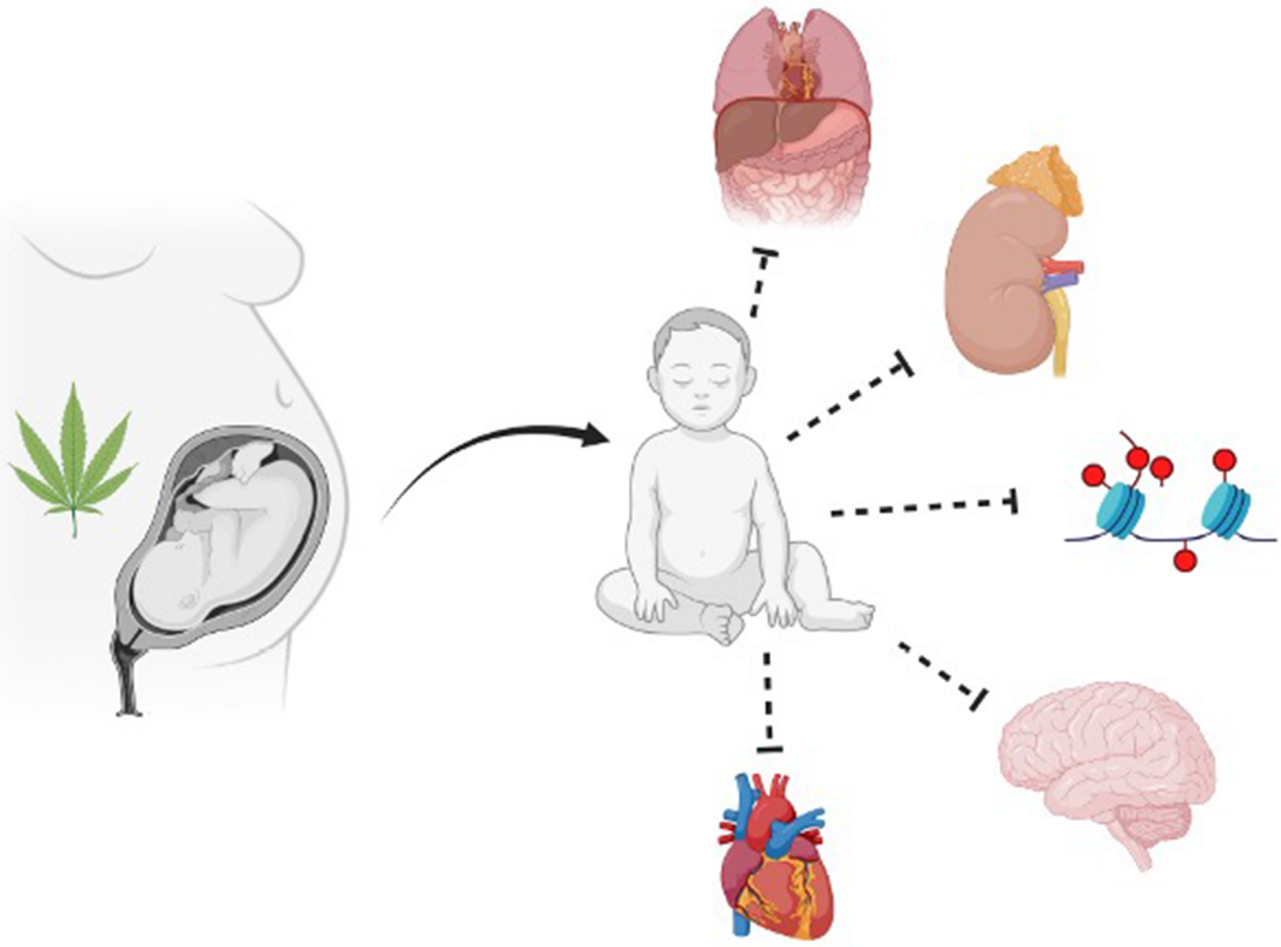
Summary of the broad impact prenatal cannabinoid exposure can have on fetal development. Multiple fetal systems can be impacted by prenatal cannabinoid exposure, including but not limited to abdominal wall, uronephrological, epigenetic, neurological, and cardiac. Figure created with BioRender.com.

The FDA-approved THC-based medications further cloud the issue of whether individuals should use these drugs during pregnancy. The available evidence suggests adverse consequences of cannabinoid exposure on female reproductive health, pregnancy, and altered trajectories of fetal development and long-term health outcomes (see Figure 1) (20, 35–37). Additionally, 48%–60% of individuals continue consuming cannabinoids during pregnancy and lactation, which affects approximately 34% of all pregnancies (19, 22, 38) The self-disclosed prevalence of cannabinoid usage during pregnancy ranges from 2% to 5% in most studies but increases to 15%–28% among young, socioeconomically challenged, urban individuals(19). However, due to the longstanding status of cannabinoids as an illicit drug, there is a dearth of well-designed unequivocal studies that assess the effects of perinatal cannabinoid exposure on fetal and placental health outcomes (22, 39). The equivocal results of many studies of perinatal cannabinoid use, the increased potency of natural and synthetic cannabis products, the increased availability of cannabinoids due to ever-increasing legalization for medical and recreational uses, and the perceived innocuousness of cannabinoids have all contributed to an increased prevalence of pregnant individuals using cannabinoid products.

During the COVID-19 pandemic, the prevalence of cannabinoid use increased among people of reproductive age, due, in part, to elevated anxiety and stress levels. Specifically, due to COVID-related stressors (e.g., loneliness-induced mental health crises, unemployment, the responsibility of homeschooling added to parental childcare, neglected prenatal care, and fear of being infected with the coronavirus), there was a significant increase in the rates of urinalysis-confirmed prenatal cannabis use among pregnant individuals in Northern California. The increased prevalence of prenatal cannabis use coincided with the dramatic increase in sale of cannabis in California at that time, as cannabis dispensaries were deemed to be essential businesses (40, 41), and identified three primary reasons why individuals use cannabinoids during pregnancy and lactation: (1) escapism or sensation seeking for pleasure, (2) management of chronic disease or amelioration of pregnancy-related symptoms, and (3) as a mechanism for coping with the vicissitudes of life. The authors concluded that the divergent reasons for pre-conceptional cannabinoid use and use during pregnancy and lactation highlights the participants' perceptions of the benefits and safety profile of cannabinoids and may be an effort to justify their usage as therapeutic to overcome the perceived stigma of perinatal drug use (41).

## Epigenetics and exogenous cannabinoids

### What effects do exogenous cannabinoids have on the developing fetus and through what mechanism are these effects potentially transmitted to subsequent generations?

The quote, “*If DNA is thought of as the cells’ bioinformatic ‘hardware’ then the epigenome can be considered its programming ’software’”* (42) is apropos. Epigenetics is the study of how heritable alterations of DNA can modify gene expression and alter the phenotype without altering the nucleotide sequence (43). Epigenetic modifications play a crucial role in guaranteeing that cells commit to a specific mitotically and meiotically inheritable phenotype and maintain the stability of the genome. The epigenetic mark-induced silencing of centromeres, telomeres, and transposons ensures that the spindle microtubules correctly connect to centromeres, decreases the incidence of recombination between repetitive sequences, and prevents translocation of transposons, which can result in mutations (43). These epigenetic modifications are thought to be the ideal candidates for the molecular mechanism by which environmental stimuli can be translated into heritable alterations of the DNA or chromatin structure. That is, epigenetic modifications are how extended perinatal (i.e., from early gestational through breastfeeding 20) cannabinoid exposure could lead to lasting effects, some of which can be transmitted to successive generations.

**Figure 2:**
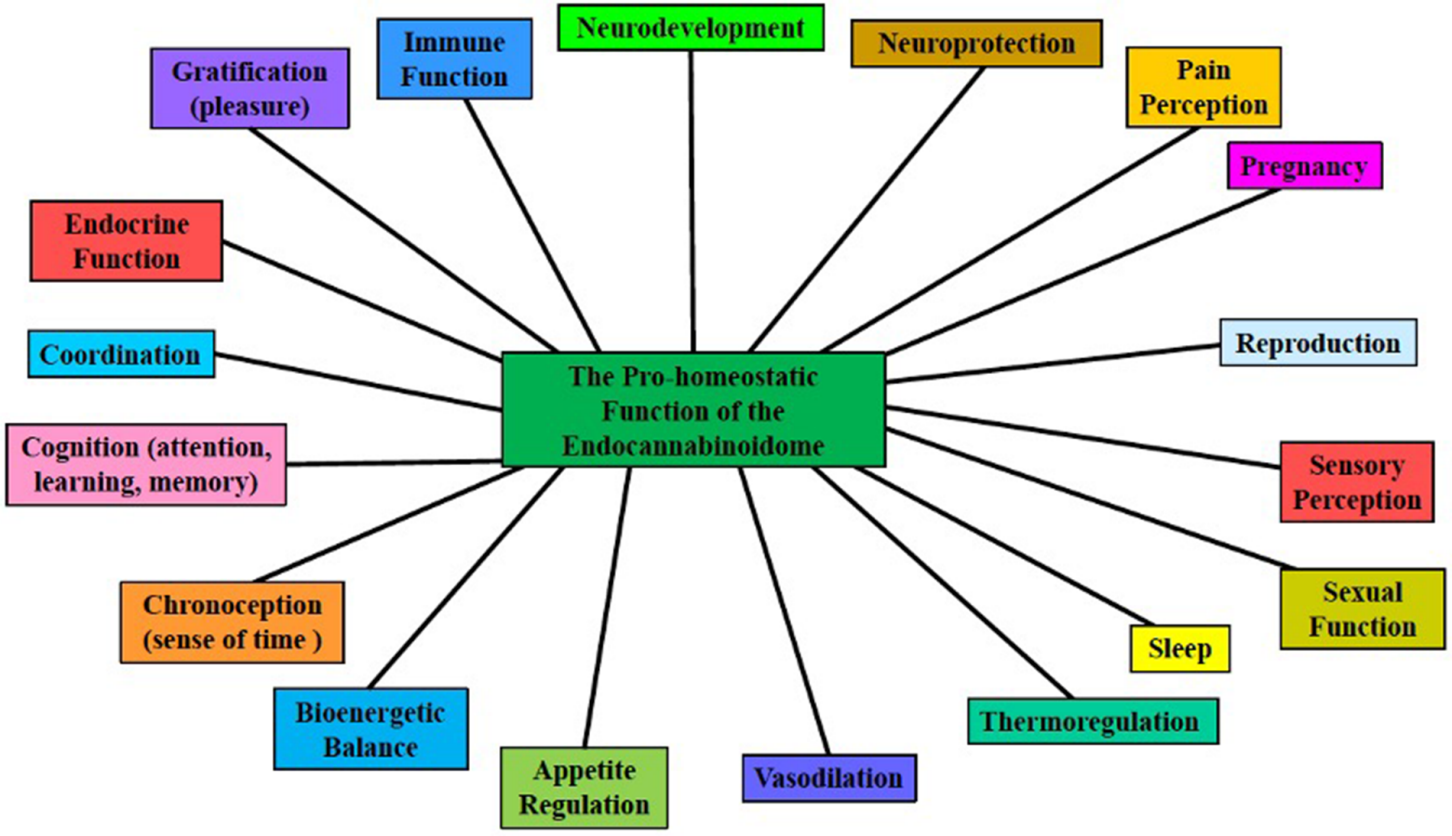
The pro-homeostatic function of the endocannabinoidome. The ubiquitous expanded endocannabinoid system (eCB, i.e., the endocannabinoidome, eCBome), which consists of many eCB-like lipid mediators, and their metabolic enzymes and molecular targets, is an overarching neuromodulatory system that plays crucial roles in neurodevelopment and neuroplasticity, in several physiological and cognitive processes, and responds to endogenous and exogenous perturbations [modified from Sun and Dey (11), Orsolini et al. (12), Di Marzo (13)].

In a review by Basavarajappa et al., several studies highlight the importance of further evaluation of CB receptor regulation by epigenetic modifications (13, 44). Di Marzo et al*.* eloquently describes the discovery of an *expanded* eCB, the endocannabinoidome (eCBome), an elaborate signaling system that participates in neuronal development and synaptic plasticity in most brain areas (13, 45–47) and is subsequently involved in the onset, progression, and symptomology of major neuropsychiatric disorders, providing a potential target for the development of novel therapeutics against these disorders [see Figure 2 (11–13)]. The eCBome is comprised of several non-eCB long-chain fatty acid amides and esters including (a) AEA and 2-AG congeners, (b) *N*-acyl-amino acids, (c) N-acylated dopaminergic and serotonergic neurotransmitters, and (d) primary fatty acid amides. AEA and 2-AG congeners can produce their effects by sharing AEA and 2-AG biosynthetic or degradative enzymes, and sometimes their receptors (13). The eCB and the eCBome mediate, and are clinical targets used to treat, several neurodegenerative diseases**,** including Alzheimer's disease, Huntington's disease, Parkinson's disease, Amyotrophic lateral sclerosis, Multiple sclerosis, Epilepsy, Glioblastoma, Stroke, and Traumatic brain injury (13).

The existence of the eCBome partially explains why some non-psychotropic CB, which modulate several eCBome proteins, are effective for the treatment of Multiple sclerosis and Epilepsy (13). Specifically, the FDA-approved, as a safe and effective tablet formulation of the THC-based medication, dronabinol (Marinol®) is an appetite stimulant for HIV/AIDS-induced anorexia and antiemetic for chemotherapy-induced nausea and vomiting, and nabilone (Cesamet®) is an antiemetic and a treatment for obstructive sleep apnea. Several other THC-based medications have been FDA-approved or are currently undergoing clinical trials. In several countries outside of the United States, nabiximols (Sativex®), an oral spray that is available for treating Multiple Sclerosis-induced spasticity and neuropathic pain, is a combination of THC and CBD. The FDA approved Epidiolex®, a CBD-based liquid formulation, for the treatment of Dravet Syndrome and Lennox-Gastaut Syndrome, which are two forms of rare, severe childhood epilepsy (29, 48).

**Figure 3:**
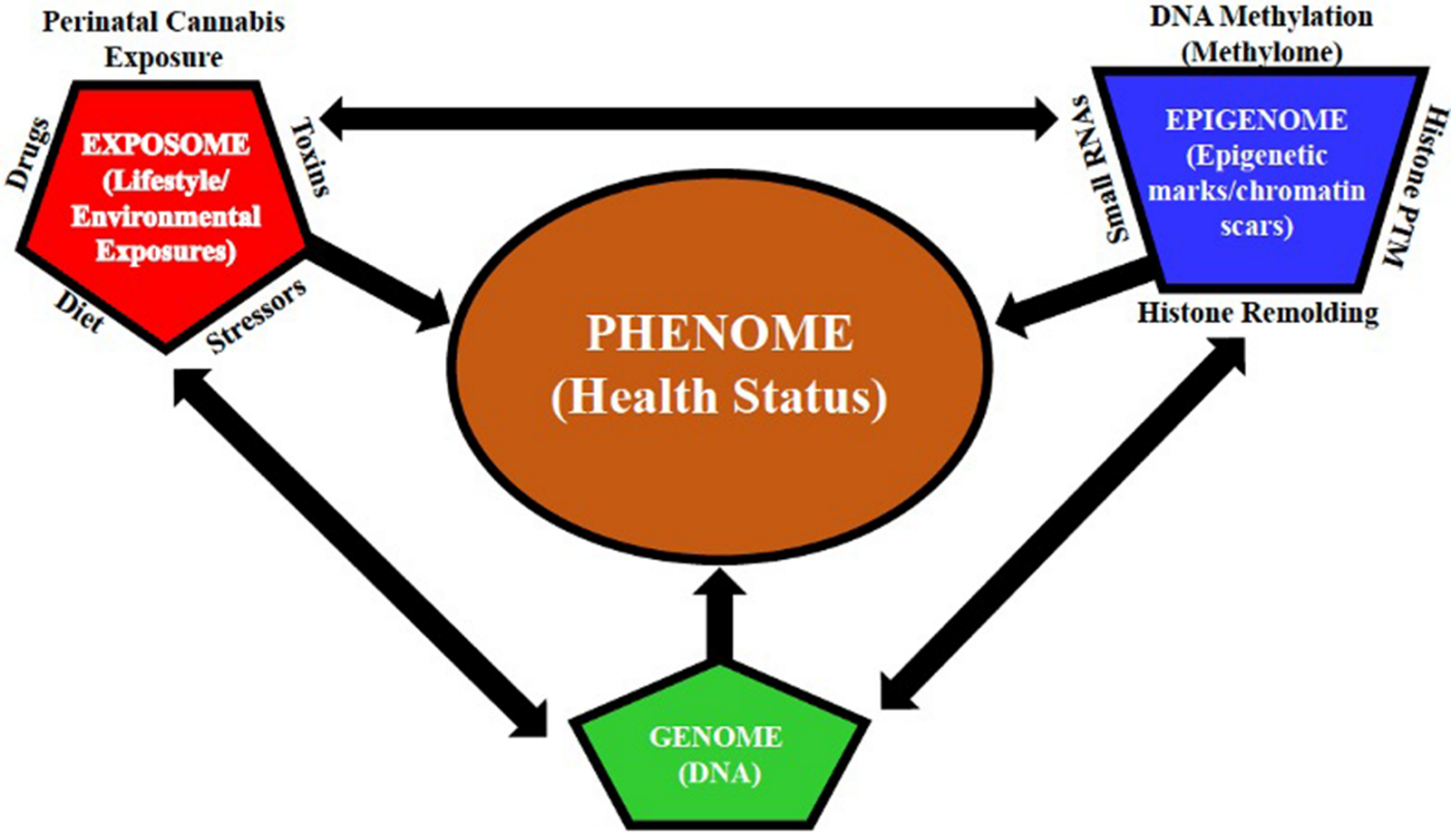
Multidimensional interactions between the genome, epigenome, and exposome determine the phenome. The multidimensional interactions between the genome (DNA), epigenome (i.e., the sum of all epigenetic marks/chromatin scars throughout the genome), and exposome (lifestyle/environmental exposures) determine the phenome (health status). Epigenetic alterations induced by cumulative lifestyle and environmental exposures (e.g., perinatal drug exposure, diet, drugs, stressors, and toxins), leading to alterations in the germline of the exposed individual, can be transmitted to the subsequent generations. The interaction between lifestyle and environmental exposures is represented by the exposome (alters the epigenome via DNA methylation (methylome), histone remodeling and post-translational modification (PTM), and gene expression regulators (small RNAs)), which, in turn, alters the genome, without altering the DNA sequence, and modulates gene expression, which, as a result, alters the phenome, which represents all phenotypic traits [modified from Paoloni-Giacobino (50)].

The precise underlying molecular mechanisms that maintain the enduring effects of perinatal cannabis exposure remain to be fully elucidated. Evaluation of fetal brain tissue with prenatal cannabinoid exposure revealed a dose dependent decrease in dopamine receptor D2 mRNA levels in the nucleus accumbens region and has been hypothesized to contribute to adverse psychiatric outcomes following prenatal cannabinoid exposure (49). This finding was replicated in a preclinical study of rats, with the reduction of D2 mRNA levels persisting into adulthood (49). Epigenetic mechanisms are, indeed, the most logical candidates to explain protracted phenotypic alterations because the epigenome (i.e., the totality of all epigenetic marks/chromatin scars throughout the genome) can translate environmental exposures (e.g., perinatal drug exposure, diet, drugs, stressors, and toxins) into stable alterations of the genome by providing a means by which perinatal cannabis exposure can alter genes and their associated phenotypes [see Figure 3 (50)]. Lifestyle choices and environmental stressors can retune the neuroepigenetic machinery, which can impact an individual's susceptibility to drugs or mental illness by exacerbating perinatal cannabinoid-induced alterations in gene expression that undergird the transition from impulsive to compulsive drug taking (i.e., addiction).

### How do epigenetic modifications contribute to a maternally cannabinoid exposed individual's subsequent susceptibility to disease or mental illness?

The “thrifty phenotype” hypothesis of the etiology of non-insulin-dependent Type 2 diabetes can be applied to perinatal cannabinoid induced fetal/prenatal programming (i.e., the hypothesis that maternal cannabinoid use during fetal development plays a decisive role in determining health trajectories across the lifespan of the offspring), and this hypothesis ignited interest in the fetal origins of adult diseases, and, in 2003, the International Society for Developmental Origins of Health and Disease was founded (51). Fetal programming represents the impact of neurodevelopmental plasticity in response to lifestyle and environmental stressors during early life and the potential adverse outcomes later in life (52). Prenatal programming is regulated by fetal genetic information (i.e., the fetal genome), which is composed of both the maternal oocyte epigenome and paternal sperm epigenome both of which underwent epigenetic modifications in response to parental lifestyle/environmental factors. These epigenetic alterations can, not only, influence the health trajectory of the fetus but the phenotype of the fetus' offspring (i.e., gametic transmission) (53).

### Why are preclinical studies paramount to our understanding of the effects of perinatal cannabinoid exposure in humans on neurodevelopment and fetal and adult outcomes in exposed offspring?

The use of non-human animal, specifically rodent, models in research is paramount to our elucidation of the effects of perinatal exposure on human development, behavior, and health, minimizing confounding variables, and establishing cause and effect relationships between the exposure and potential epigenetic alterations. For example, a recent study by Lee, et al. was able to demonstrate that prenatal cannabinoid exposure resulted in significantly smaller hearts size relative to body weight in a rat model with postnatal cardiac remodeling and impaired cardiac function (54). Indeed, a causal inferential modelling and space-time statistical analysis showed a strong bivariate relationship of prenatal cannabinoid exposure and cardiac anomalies (55), thus stressing the need for continued investigations into the etiology of altered development. Preclinical animal studies allow the interrogation of the molecular consequences of long-term cannabinoid use (i.e., epigenetic alterations) that could potentially perpetuate abnormal gene expression and the associated behavioral consequences (56–59) Given that over 50% of all pregnancies in the United States are unintentional (i.e., potential exposure of both parents to cannabinoids 60) it is important to highlight the main consequences of perinatal cannabinoid smoking in such a profound manner that it will make an indelible mark on the psyches of women and men of reproductive age, especially, members of historically marginalized and urban populations, whose communities are being inundated with high potency, easily available natural and synthetic cannabis products, so that those who are contemplating having a baby will not dare use cannabinoids before conception, during and after pregnancy and lactation, or during their child's adolescence. With this goal in mind, we will focus on prenatal cannabinoid exposure by summarizing in chronological order the preclinical studies on the effects of PCE in rats and mice [see Supplementary Table S1 (61, 62)]. For the sake of brevity and because of the detailed nature of the table, we will not recap the experiments included in the table herein.

## Contributions of clinical studies: what have they revealed about prenatal cannabinoid exposure?

Human studies of cannabinoid exposure during pregnancy that were performed in the 1980′s, when prevalence of use and THC potency were lower, partially support disturbances in fetal neurodevelopment, increased risks of stillbirth, increased incidence of fetal growth restriction, and long-term adverse neurodevelopmental outcomes (35). Additionally, retrospective studies that control for confounding variables (e.g., polysubstance use, inadequate sample size, unhealthy lifestyle) are scarce and, thus, yield contradictory results, as many of these studies rely solely on uncorroborated patient self-report, which increases recall bias, precluding a definitive causal relationship between negative developmental outcomes and perinatal cannabinoid use (19, 22, 63). Unlike the standardization of distinct types of alcohol pours by volume, there is no such standardization for cannabinoid potency because different strains of marijuana plants and the route of administration vary in potency. Currently, there is no dependable method to accurately quantify in biological samples the amount of cannabinoid used. Therefore, it should be a global imperative to develop and validate reliable analytical methods for cannabinoid screening (22, 64).

To date, there have been six longitudinal studies that investigate the developmental correlates of prenatal cannabis exposure (PCE). These include the Ottawa Prenatal Prospective Study (OPPS), Maternal Health Practices and Child Development Study (MHPCD), Generation R study (GenR), Adolescent Brain Cognitive Development Study (ABCD), Lifestyle and Early Achievement in Families study (LEAF) and the Norwegian Mother and Child Cohort Study (MoBa). In addition to these single cohort studies, the Environmental Influences on Child Health Outcomes (ECHO) program contains data on PCE. The study characteristics, enrollment criteria and outcome measurements of these longitudinal cohort studies should inform their findings given the everchanging landscape of cannabis use, potency, and social acceptability.

Epidemiological studies using sophisticated space-time and causal inferential statistical analyses have revealed multiple concerning associations, including higher congenital anomalies (65, 66) such as neurological anomalies (67), body wall anomalies (68), and uronephrological congenital anomalies (69). Specifically, the defects include severe microcephaly, craniosynostosis, microphthalmos, anencephalus, hydrocephalus, neural tube defects, omphalocele, diaphragmatic hernia, gastroschisis, bilateral renal agenesis, multicystic renal disease, hydronephrosis, and congenital posterior urethral valves (67–69). Additionally, rates of autism in the United States appear to be associated with PCE, which persists after controlling for other major covariates (70), although large studies are needed to confirm this relationship.

Together these analyses and longitudinal studies have demonstrated associations between PCE and congenital anomalies as well as negative neurocognitive outcomes in children from infancy to late adolescence. The most replicated associations are deficits in short term memory, impulse control, and attention deficiencies. A summary of clinical studies is presented in Table 1. For the sake of brevity and because of the detailed nature of the table, we will not recap the studies included in the table herein.

**Table 1:** Summary of selected clinical studies on the effects of perinatal cannabinoid exposure in infants to young adults.

Study/trial name	Age range	Participants	Results	References
MHPCD	Infant	519	• Few significant effects of cannabinoid use during pregnancy on birth weight, head or chest circumference, gestational age, or growth retardation • Small significant negative effect during first two months of pregnancy on birth length resulting in shorter infants, but no reduced birth weight • Small significant positive effect during the third trimester on birth weight	1. Day N, Sambamoorthi U, Taylor P, Richardson G, Robles N, John Y, Scher M, Stoffer D, Cornelius M, Jasperse D 1990
LEAF	Infant	325	• Frequency of Small for Gestational Age (SGA) less than 10th percentile was higher with cannabinoid use (35%) than other substances • Frequency of SGA less than 5th percentile was higher with cannabinoid use (30%) than other substances	2. Abdelwahab M, Klebanoff MA, Venkatesh KK 2022
OPPS	Birth—early adolescence	140	• Head circumference were smallest for heavy cannabinoid user, intermediate for mild/moderate users, and largest for non-users with this trend being statistically significant in 9–12-year-old children (*p* < 0.05) • 1–4 years: average weight and height, but not PI, greater for the children born to heavy cannabinoid users than the other 2 groups, but differences not significant • Birth of infants to heavy cannabinoid users were lightest of 3 groups, but at age 1 were the heaviest with significantly more weight than the children of the other groups (*p* < 0.05) • Female children in heavy cannabinoid users were significantly lighter than males at 12 months of age (*p* < 0.01)	3. Fried et al. (71)
Generation R	18 months (girls)	4,077	• Gestational exposure to cannabinoids is associated with behavioral problems in early childhood but only in girls and only in the area of increased aggressive behavior and attention problems • Exposure to cannabinoids led to increased scores on the aggressive behavior scale of the CBCL in girls, but not in boys • Paternal cannabinoid use was not associated with aggressive behavior in either boys or girls • Girls born from PCE had an increased risk of developing aggressive behaviors, but it was not statistically significant • Cannabinoid exposure led to increased scores on attention problems scale of the CBCL in girls, but not in boys • Girls had an increased risk of developing attention problems (*p* = 0.01) • No association found between PCE in girls or boys and anxious or depressive symptoms	4. Marroun HE, Hudziak JJ, Tiemeier H, Creemers H, Steegers EAP, Jaddoe VWV, Hofman A, Verhulst FC, Van de Brink W, Huizink AC 2011
OPPS	36–48 months	134	• 36-months: Moderate group had better motor skills than heavy and little use groups • 36-months: Motor scores higher for moderate group compared to the heavy or little use groups • 36 months: Quantitative scores lower in heavy group than other two groups • 48-months: Heavy group had lower mean scores on discriminating variable from non-users and moderate group • 48 months: Heavy group had poorer memory skills then moderate or little use groups	5. Fried and Watkinson (72)
MHPCD	3 years	655	• Association with lower efficiency and maintenance, more awake time, and a higher number of arousals which indicate early subtle differences in brain development • Effects of PCE on composite score, and the short-term memory and verbal reasoning subscales of the Stanford Binet Intelligence Scale • Relation between PCE and abstract/visual reasoning in a cohort of poly-substance users	6. Day NL, Richardson GA, Goldschmidt L, Robles N, Taylor PM, Stoffer DS, Cornelius MD, Geva D 1994
LEAF	3.5 years	63	• 15 classified as PCE, but only 2 self-reported cannabinoid use indicating a large portion would have been misclassified • Caregivers reported on children's EF and problem behaviors • Children with prenatal cannabinoid exposure (PCE) had more sleep-related problems, withdrawal symptoms, and externalizing problems, including aggressive behaviors and oppositional defiant behaviors • Children with and without PCE did not differ in terms of executive functioning • Children with PCE score significantly worse on Bayley puzzle task; 31% less likely to be able to complete the task • Children with PCE who engaged with the doll during the Bobo task displayed significantly more aggressive behaviors towards the doll such as fisted hits to the face	7. Murnan et al. (73)
OPPS	72 months (6-year-old)	126	• Increased omission errors in the vigilance task, possibly reflecting a deficit in sustained attention • Total correct on vigilance task was negatively correlated with cannabinoids • Number of omissions on vigilance task and Impulsive/Hyperactive scale were positively correlated • Change in correct classification rates for the three cannabinoid groups were 72.5% to 71.7% • Examination of the delay and vigilance scores across successive temporal blocks revealed no statistically significant differential variation across cannabinoid groups • Total correct and omissions scores of the vigilance task, a nonsignificant between groups effect and nonsignificant interaction appear to contribute substantively to the discriminant function • Number of errors increased between the first and last block by 13% with children in the heavy category compared to 3–4% in moderate and light categories	8. Fried PA, Watkinson B, Gray R, 1992
MHPCD	6 years	608	• Second-trimester cannabinoid use predicted more commission errors (impulsive responding) and fewer omission errors (measure of inattentiveness) • Commission errors had no significant effects on prenatal alcohol, cocaine, or tobacco exposure for any trimester in comparison to cannabinoid exposure • Errors of commission significantly predicted by composite score of the Stanford-Binet, gender, and presence of a male in the household	9. Leech SL, Richardson GA, Goldschmidt L, Day NL 1998
MHPCD	6 years	648	• Significant nonlinear relation between cannabinoid exposure and child intelligence • First-trimester heavy users lead to lower verbal reasoning scores on the Stanford-Binet Intelligence Scale • Second-trimester heavy users predicted deficits in composite, short-term memory, and quantitative scores • Third-trimester heavy users were negatively associated with quantitative score • Significant predictors included maternal IQ, home environment, and social support • No significant differences on abstract/visual reasoning	10. Goldschmidt L, Richardson GA, Willford J, Day NL 2008
Generation R	7–10 years	5,903	• Maternal cannabinoid use before and during pregnancy resulted in offspring showing externalizing problems, but not internalizing • Paternal cannabinoid use resulted in offspring showing externalizing problems but not internalizing problems • Prenatal cannabinoid exposure is associated with behavioral problems, but not emotional problems	11. El Marroun et al. (74)
ABCD	9–10 years	11,489	• 655 participants prenatally exposed to cannabinoids • Cannabinoid exposure only before and after maternal knowledge of pregnancy were associated with greater offspring psychopathology characteristics (PLEs and internalizing, externalizing attention, thought and, social problems), sleep problems, and body mass index as well as lower cognition and gray matter volume (GMV) [all false discovery rate (FDR)-corrected *p* < 0.03] • Exposure after knowledge of pregnancy resulted in lower birth weight, total intracranial volume, and white matter volumes (FDR-corrected *p* < 0.04) • Exposure after knowledge of pregnancy including confounding covariates resulted in greater PLEs and externalizing, attention, thought, and social problems (FDR-corrected *p* < 0.02) • Exposure only prior to knowledge of pregnancy did not differ from no exposure (FDR-corrected *p* > 0.70) • Prenatal cannabinoid exposure and correlated factors result in a greater risk for psychopathology during middle childhood	12. Paul SE, Hatoum AS, Fine JD, Johnson EC, Hansen I, Karcher NR, Moreau AL, Bondy E, Qu Y, Carter EB, Rogers CE, Agrawal A, Barch DM, Bogdan R 2020
ABCD	9–10 years	11,489	• 224 participants were prenatally exposed to cannabinoid after knowledge of pregnancy • PCE had statistically significant increased attention problem scores compared to the control • Only significant fixed effect variable was parental psychopathology with increasing ASR total problem scores being associated with higher attention scores • CBCL Externalizing symptoms and total problems were significantly higher in participants with PCE compared to controls • CBCL Social problems were not significantly different • No significant differences in any neurocognitive tasks • No significant differences in BOLD signal activation patterns	13. Cioffredi at al. (75)
OPPS	9–12 years	131	• Strongest negative associations were observed from the Category test, the WISC Block Design and Information, and the total number of responses on the Gordon Delay task. None remained statistically significant after controlling covariates • Significant relationship found between maternal cannabinoid use and variables associated with aspects of executive function (*p* < 0.01) • First discriminant: 72% of the total discriminatory variance and was used to distinguish cannabinoid users from the control group ○ Means- control: −0.29, infrequent/moderate: 0.68, and heavy: 0.71 • Main discriminating measures were Category test: 0.49, Gordon Delay total number of responses: 0.34, WISC-Block Design: −0.30, WISC-Picture Completion: −0.30, Gordon Delay efficiency ratio: −0.29, where cannabinoid users scored more poorly than the control • Only the mother's age at the time of pregnancy was associated with discriminant scores	14. Fried et al. (76)
MHPCD	10 years	636	• Prenatal cannabinoid use is significantly related to increased hyperactivity, impulsivity, and inattention symptoms as measured by Swanson, Noland, and Pelham (SNAP), increased delinquency as measured by Child Behavior Checklist (CBCL), and increased delinquency and externalizing problems as measured by Teacher's Report Form (TRF) • First-trimester cannabinoid use remained significant for predicting the attention scale of the SNAP after controlling for other risk factors (*p* < 0.01) • Third-trimester cannabinoid use remained significantly associated with higher scores on hyperactivity (*p* < 0.001), attention (*p* < 0.01), and impulsivity (*p* < 0.01) • Second-trimester cannabinoid use was associated with fewer internalizing behavior problems on CBCL and remained significant after adjusting for covariates (*p* < 0.05) • Children prenatally exposed to heavy cannabinoid use were at relative risk for scoring above the cutoff score on the CBCL delinquency subscale was 2.4 times that of a child not exposed (*p* < 0.01)	15. Goldschmidt et al. (77)
OPPS	13–16 years	152	• 13 years: impulsivity scores were statistically significantly different across the two smoking groups of nonsmokers and smokers (*P* < 0.05) • Both attention components labeled as encode/retain and short form of the WISC-III were associated with maternal smoking (*p* < 0.001) • Impulsivity factor also related to prenatal smoking • Maternal cannabinoid use associated with poorer performance on stability • Heavy paternal cannabinoid use was associated with factor scores reflecting less consistent reaction time over blocks and more omissions	16. Fried and Watkinson (78)
OPPS	13–16 years	145	• Significant association between Abstract Design latency and the cannabinoid groups with the subjects born to heavier users displayed slower response times • Performance on Peabody Spelling negatively associated with maternal cannabinoid use	17. Fried et al. (77)
MHPCD	16 years	47	• Behavioral and fMRI data revealed no associations between PCE and task accuracy, speed of processing, or activation in key brain regions that are associated with attention networks • Subtle differences in brain function associated with PCE were not detected	18. Willford JA, Singhabahu D, Herat A, Richardson GA 2018
MHPCD	14 years	829	• 46% of adolescents smoked a cigarette, 35% drank alcohol and 30% tried cannabinoids • Adolescents with PCE did less well on coding, block design, and maze tests on the WISC-II	19. Day NL, Leech SL, Goldschmidt L 2012

### The Ottawa prospective pregnancy study (OPPS)

Conducted in Ottawa, Canada the OPPS enrolled 698 pregnant individuals between 1978 and 1982 (71). Individuals were recruited from prenatal clinics at the major hospitals in Ottawa, and those with prenatal use of cannabinoids, alcohol, and tobacco were included as well as individuals with no prenatal use (79). The average age of pregnant individuals at enrollment was 29 years, mean family income comparable to the mean family income in the Ottawa metropolitan area, and only 4% had less than a high school education while 70% had more than a high school education (80). Prenatal drug exposures were measured by participant report at each of these interview time points including alcohol, cannabis, and tobacco. Cannabis was measured in marijuana “joints” per week and categorized into non-users, light users (≤1 joint per week or those exposed to second-hand cannabis smoke), moderate users (2–5 joints per week), and heavy users (>5 joints per week).

After the infants were born and initial studies completed, a subset of the original cohort was selected for follow up. This included 140 individuals who used cannabis during pregnancy, or individuals who were heavy users of alcohol or nicotine during pregnancy. Additionally, 50 individuals who reported no use were randomly selected to serve as controls (72). Follow up of this cohort spans 22 years since infants were born. Executive functioning deficits, including difficulty with impulsivity and attention, have been noted through adolescence (76, 78). Although the young adult imaging studies are much smaller in size, the follow up cohort established after birth maintained 80%–83% retention through mid-adolescence (78, 81).

### The maternal health practices and child development project (MHPCD)

In 1982, the faculty at the Magee-Women's Hospital in Pittsburg, Pennsylvania began enrolling pregnant individuals in the MHPCD. Potential participants, at least four months into their pregnancy, were randomly selected from outpatient obstetric clinics. All individuals who reported cannabis use as more than two joints a month during their first trimester were chosen for continued follow up, and an equal number of participants who reported less use were randomly selected from the remaining subjects. They completed follow up intervals assessing drug use in their 3rd trimester and birth hospitalization (82). Unlike the OPPS cohort, the MHPCD cohort had a more diverse population of participants with lower average age (22 years) and income (69% less than $5,000 per year) (83).

Alcohol, cannabis, and tobacco exposures were assessed with questionnaires administered prospectively throughout pregnancy. Cannabis quantity was measured in marijuana joints per day, and like OPPS, the use of hashish was considered to count as more cannabis than marijuana and was multiplied by three to “convert” to joints. After the birth assessment, children in the MHPCD cohort were followed up at multiple time points. At 10 years of age, PCE was significantly related to abnormal executive functioning with increased hyperactivity, impulsivity, and inattention symptoms in children with exposure (77). At 22 years of age, most of the original cohort (69%, 524 individuals) was retained for continued assessment (84).

### Generation R (GenR) study

The GenR study is a population-based prospective cohort study based in Rotterdam, Netherlands. Pregnant individuals and their partners residing in Rotterdam with delivery dates between April 2002 and January 2006 were eligible for participation. A total of 9,778 pregnant individuals were enrolled, and 1,232 participants were enrolled in the subgroup who would participate in more detailed assessments of health and development during the preschool years of the children. This cohort includes participants whose average maternal age at enrollment was 30.5 years (older than those previously mentioned), and a less diverse population with most participants being Dutch or other European ancestry and having at least secondary school education (89%) (85).

Cannabis exposure was measured via participant report during the first trimester with a question asking about cannabis use in the past 3 months (to capture before pregnancy) and had answers “No,” “Yes until I knew I was pregnant” and “Yes I still use.” Additionally, THC metabolites were evaluated in the third trimester using urine testing (74). Unlike OPPS and MHPCD, GenR was not specifically aimed to assess the impact of prenatal drug exposures, but rather to describe normal and abnormal growth and development as well as identify environmental, biological, and social factors, which influence growth and development. In addition to questionnaire and objective assessments this study included biospecimens such as maternal and child blood and urine (85). Interestingly, a sex effect has been noted thus far at age 18 months, with girls having behavioral issues not observed in boys (86) after controlling for parental education, national origin, and parental psychopathology.

### The lifestyle and early achievement in families (LEAF) study

With an ambidirectional study design, the LEAF study adds prospective developmental follow up to an existing cohort study at the Ohio State University in Columbus, Ohio. The initial study enrolled participants while they were pregnant in 2010 and continues to enroll at the time of this publication. Initial inclusion criteria for pregnant participants include age 16–50 years, English speaking, and intent to delivery at the Ohio State Medical Center (87). The prenatal protocol includes a question about cannabinoid use during pregnancy. Medical records from the delivery hospitalization, including drug screen results, were obtained at the conclusion of the initial cohort study. Subsequently, a study sample was obtained from those who agreed to be contacted after the original study concluded, and whose children would be old enough for follow-up (71% of the original cohort). This resulted in a cohort of 361 children who were born between 2010 and 2016. Within this follow up cohort, the mean age during pregnancy was 26 years, 31% of the cohort had less than a high school education while 38% had at least some education after high school. The majority (63%) of the sample was African American (87).

Measurement of prenatal cannabis exposure in this cohort is multifaceted. Participants were considered cannabis exposed if there was prenatal self-report of cannabis use, if it was noted in the obstetric medical record, or if any of the urine samples collected during pregnancy had THC concentrations >15 ng/ml (87). There are 117 participants who meet these criteria for prenatal cannabis use. At 3.5 years of age, the PCE children had more sleep-related problems, aggressive behaviors, and oppositional defiant behaviors (73) after controlling for child race, child sex, prenatal tobacco exposure, maternal/caregiver marital status, household income, and maternal/caregiver executive functioning.

### The Norwegian mother and child cohort study (MoBa)

Perhaps the largest pregnancy cohort study evaluating prenatal cannabinoid exposure is the Norwegian Mother and Child Cohort Study (MoBa) in which 114,000 children from Norway have been followed for up to 13 years of age, beginning at birth. Enrollment was open to all pregnant individuals in Norway between July 1999 and December 2008. A total of 41% of pregnant Norwegians agreed to be part of the study. Retention through pregnancy was maintained at 95% of those enrolled and fell to 77% by 18-month assessments (88). The data includes biosamples (whole blood, plasma, and urine) collected from pregnant individuals and their partners during pregnancy as well as the pregnant individual and their child at delivery (89). Of the total sample, 9,312 pregnant individuals reported lifetime cannabinoid use resulting in 10,373 pregnancies. However, in only 272 pregnancies had participants used cannabinoids *during* pregnancy. Cannabinoid use in this large study was self-reported and included only hashish, as it is historically the only form of cannabinoids used in Norway (90).

### Adolescent brain child development study (ABCD)

ABCD represents the largest cohort study on child brain development in the United States to date. Baseline assessments began in 2016 when children were 9–10 years old and is planned to have continued assessments for 10 years (91). The study consists of 11,880 children and includes multiple batteries of assessments spanning health and behavior, including mental health, neurodevelopment and cognition, daily activities, exposures, substance use, and neuroimaging (92). Participants were recruited from 21 sites across the country with the goal of mirroring the demographics of the United States. Prenatal exposures, including cannabinoids, were assessed at time of enrollment, and generally categorized into use before knowledge of pregnancy and after. Caregivers were asked 2 questions: “Before you (or the biological mother)” and “Once you (or the biological mother) knew you were (she was) pregnant were you (was she) using any of the following?” The list that followed included tobacco, alcohol, marijuana, other illicit drugs as well as prescription drugs. Possible answers included, “yes,” “no,” and “don't know.” Duration and trimester of use was not collected. For those who reported use, daily frequency was collected by parent or caregiver retrospective report. For children with PCE at 9–10 years of age, significant increases in attention problems were noted (75).

### Environmental influences on child health outcomes (ECHO) program

The Environmental Influences on Child Health Outcomes (ECHO) Program is focused on observational research, with support for individual cohort science but more importantly brings separate cohorts together into one large cohort consisting of children, mothers, and fathers (93). The children are followed long-term with data from over 105,000 participants and over 64,000 children as of April 2023 (93). This organization consists of 44 sites across the United States and Puerto Rico (94). Exposure during pregnancy is generally confirmed via self-report. There is an opportunity to use study samples such as urine and measure metabolites to confirm exposure (95). With an abundance of data that continues to be collected, findings from this observational collaboration are regularly being published.

#### Why should clinicians preemptively caution individuals of child-bearing age to take a pregnant pause from using cannabinoids during their pregnancies?

**Figure 4:**
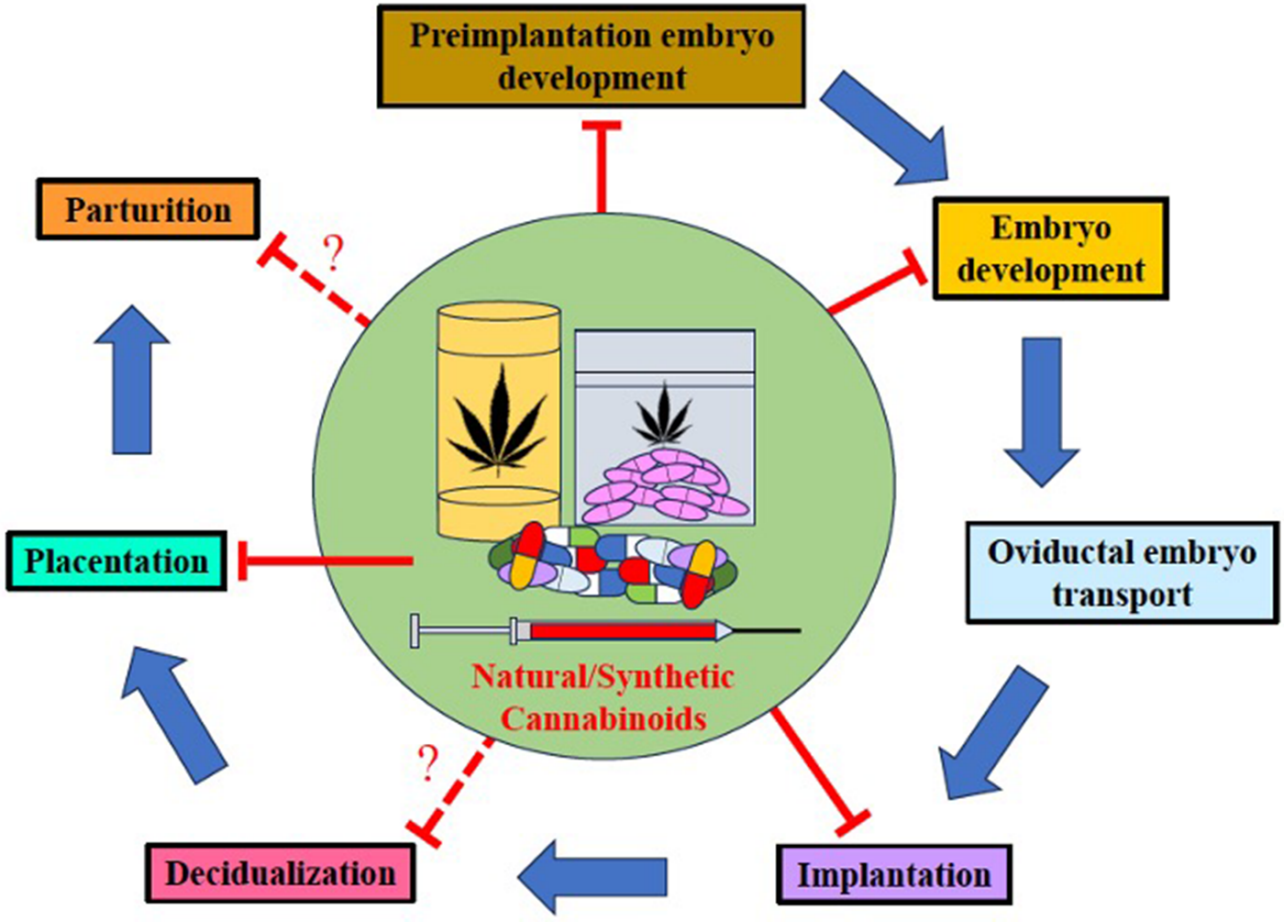
Natural and synthetic cannabinoids can target pregnancy events. Both natural and synthetic cannabinoids target multiple stages of pregnancy. Perinatal use of natural and synthetic cannabinoids disturbs several events in pregnancy including preimplantation embryo development, embryo development, oviductal embryo transport, implantation, placentation, and perhaps, decidualization and parturition [modified from Sun and Dey (11)].

Since many pregnant individuals opt to use natural or synthetic CB during the perinatal period to alleviate pregnancy-related symptoms, pre-existing maladies, stress, and for its euphoric effects (41), clinicians should caution individuals of child-bearing age of the following: (1) In rats, THC and the primary metabolite, 11-OH-THC, readily cross the blood-brain-barrier to exert significant effects on the brain (96); (2) THC crosses the maternofetal placental barrier in humans and other mammals (11, 97, 98) to bind with CB1R and affect fetal growth and development; (3) In humans, rats, and mice, canonical and non-CB receptors/channels are expressed early in placental tissues(15); (4) The eCB is present and fully functional in early fetal development (99) and exists ubiquitously in the brain from the early embryonic stage through the postnatal stage, playing a pro-homeostatic role in early embryonic and prenatal neurodevelopment (13); (5) In humans, inhaled cannabinoids can be transferred into breast milk (100); (6) Several components of the eCB have been identified in the rat embryo, follicular fluid, ovaries, the placenta, and the uterus (14, 15); and (7) In mice, synthetic cannabinoids can target multiple sites and stages of pregnancy [See Figure 4 (11)]. Therefore, it is reasonable for clinicians to hypothesize that perinatal cannabinoid exposure could potentially have detrimental consequences on fetal neurodevelopment and outcomes.

Although the effects of cannabinoids on human fetal neurodevelopment remain to be unequivocally elucidated, some studies suggest that prenatal cannabinoid exposure may be linked to subsequent deficits in attention, learning and memory, critical thinking skills, and behavioral issues in exposed offspring (see Supplementary Table S1). These potential deleterious effects of cannabinoid use during pregnancy on offspring neurodevelopmental outcomes and cognition, which initially may be subtle or undetectable for years after parturition (i.e., the first hit) and manifest only after the second hit (e.g., maternal stressor, and postnatal and caregiver exposures) (35).

## Summary and future directions

There are many problems with the existing human studies on perinatal cannabinoid exposure. Many of these studies were conducted over 40 years ago when cannabinoid potency was less, and its prevalence was lower. Several studies are contradictory and limited in breadth, contain experimental design flaws, or are fraught with confounding variables. For instance, retrospective studies, dependent on unreliable study participant self-disclosure (i.e., increased recall bias), were without quantitative urine or meconium assays to accurately assess exposure. Participants' lifestyle choices, which introduced concurrent disease states or polysubstance use, confounded many studies. Lastly in studies with low statistical power (overestimates of effect size and low reproducibility of results) and no standardized outcome measures (101, 102), the conditions preclude the determination of a cannabinoid-specific causal effect.

Given the absence of cause-and-effect evidence of perinatal cannabinoid exposure-induced detrimental fetal neurodevelopment consequences and outcomes, the authors suggest that pregnant individuals practice a cautious approach by taking a pregnant pause from using cannabinoid products (35). Additionally, pregnant individuals or those contemplating pregnancy should cease using cannabinoids for medicinal purposes when an alternative therapy with better pregnancy-specific safety data exists (19).

## Author note

Through consulting with a University of New Mexico Health Sciences Librarian (Alexis Ellsworth-Kopkowski) searches were run through PubMed, Web of Science, and Google Scholar. The PubMed search was completed on May 23, 2023, utilizing the keywords: prenatal cannabis exposure and outcomes. These results were filtered to English with the publication years of 2017–2023. The Web of Science search was completed on June 16, 2023, utilizing the keywords: prenatal cannabis exposure and outcomes and human. These results were filtered to English with the publication years of 2017–2023. The Google Scholar search was completed on June 16, 2023, and was deduplicated against the PubMed and Web of Science citations. The keywords prenatal cannabis exposure and outcomes were utilized. These results were filtered to 2019–2023.
