# Supplementary material for: Prenatal cannabinoid exposure: why expecting individuals should take a pregnancy pause from using cannabinoid products
Source: Front Pediatr. 2023 Oct 11;11:1278227. doi: 10.3389/fped.2023.1278227 (PMC10598870; doi:10.3389/fped.2023.1278227)
Supplement: Supplementary file 1 [file Table1.docx]

| **Table 1: Chronological Summary of Selected Preclinical Studies on the Effects of Perinatal Cannabinoid Exposure (PCE) in Rats and Mice (from Campolongo et al., 2011; M. Mulligan & K. M. Hamre, 2023)** | | | | |
| --- | --- | --- | --- | --- |
| **Drug (Dose, Route) or Vehicle** | **Rodent Model** | **Time Administered/ Time Tested** | **Results** | **References** |
| **Experiment 1:**  Delta-9-tetrahydrocannabinol (THC; 0.01, 0.1. 1, 10 mg/kg/day, subcutaneous (s.c.)) or Olive oil  **Experiment 2:**  THC (25, 50, 75, 100 mg/kg/day, s.c.)  **Experiment 3:**  THC (50, 100, 200 mg/kg/day, s.c.)  *Dams sacrificed via ether overdose on day 21 & examined for alterations in the weight of major internal organs & fetuses weighed & inspected for gross malformations.* | Long-Evans  Rats | Gestational Day (GD) 1-20/Postnatal Day 21 (P21) | - No effect on incidence of external malformation immediately postpartum or at weaning with THC (> 100 mg/kg) - No effect on the number of stillborn progeny or tendency for dams to lose entire litter. - Decreased litter size for THC (100 mg/kg)-treated dams, indicating embryotoxicity. - Increased pregnancy length (23-24 days versus 21-22-day gestation) in dams treated with higher THC doses. - Decreased pup postnatal survival rate in THC (> 50 mg/kg)-treated dams & an increased number of pups died, from starvation, by 1-3 days; pups attempted to nurse but no milk was visible in pups’ stomachs. - Cross-fostering of THC offspring to control females & control progeny to THC-treated dams: pups of THC dams survived & fostered control pups did poorly, confirming that THC-treated dams were not lactating. - At weaning, surviving pups normalized in size & appearance & decreased litter size for THC (50, 75 & 100 mg/kg)-exposed pups. - Decreased body weight during gestation in THC (> 10 mg/kg)-treated dams & decreased body weight with THC (10 mg/kg) during the 3^rd^ trimester & decreased weight from day 3 onward with THC (> 25 mg/kg) - Decreased maternal weight, litter size & alterations in maternal organ size (expressed as organ weight: body weight ratios) - Increased in relative mass of the maternal heart, adrenal glands & thyroid & decreased liver size & no effect on other organs. - No effect on the incidence of congenital defects or weight in day 21fetuses | 1. Borgen LA, Davis WM, Pace HB, 1971 |
| THC (25, 50, or 100 mg/kg/day, s.c.) or Propylene glycol | Sprague-Dawley Rats | GD6-15/~ P20 | - Decreased maternal weight gain & fetal weight at THC (50 mg/kg) - No gross malformations but some rudimentary14th rib abnormalities & spongy spinal cords | 1. Banerjee BN, Galbreath C, Sofia RD, 1975 |
| THC (5,15, 50, or 150 mg/kg/day, oral gavage - per os – (p.o.) or Sesame oil | CD-1 Mice | GD6-15/~ P21 | - No effect on maternal weight gain, prenatal mortality, fetal weight, or gross external, internal & skeletal morphologies in THC-exposed mice pups (pinkies) | 1. Fleischman RW, Hayden DW, Rosenkrantz H, Braude MC, 1975 |
| THC Smoke (~3.3 mg/kg/day, per inhalation – (p.i.) or smoke from material minus cannabinoids  Key: Pups born to Control dams & raised by Experimental dams (C-E)  Pups born & raised by Control dams (C-C)  Pups born to Experimental dams & raised by Control dams (E-C)  Pups born & raised by Experimental dams (E-E) | Wistar Rats | GD1-19/P7 | - No effect on litter size, gestational length, live births, or morphology at birth but increased number of males versus females/litter of THC exposed dams - Decreased birth weights in males & females. - Decreased body weight of pups born to control dams but raised by THC-exposed dams (C-E) versus pups born to & raised by control dams (C-C) during the last 15 days of nursing, but weights normalized to controls 1 month after weaning. - Pups born to THC-exposed dams but raised by control dams (E-C) initially weighed 6-8% < control pups & by weaning the difference was 3% & normalized to controls after 1 month. - E-C & E-E groups had decreased activity at P9 & had delayed incisor eruption & eye opening. - When pups were chronically injected with THC starting at P100, the C-E pups developed tolerance sooner than controls whereas the E-C & the E-E groups took longer to develop tolerance. | 1. Fried PA, 1976 |
| THC (2 mg/kg/day, p.o.) or Polyvinylpyrrolidone in Distilled water | Sprague-Dawley Rats | GD3-22/P21 & 90 | - Chemical analysis of pup tissues revealed an average THC level of ~ 20 ng/g obtained via placental transfer. - Decreased acquisition of a passive avoidance response at P21 but normalized by P90. - THC-exposed pups forced control pups to withdraw from a push tube in 67% of tests at P21 & 94% of the tests at P90 | 1. Vardaris RM, Weisz DJ, Fazel A, Rawitch AB, 1976 |
| **Experiment 1:** THC or cannabinoid exposure (Crude Marijuana Extract, CME) 0.5, 1.5, 5.0 mg/kg/day, p.o.) or Sesame oil  **Experiment 2:** THC or cannabinoid exposure (CME) (0.5, 1.5, 5 mg/kg/day, p.o.) or Sesame oil  **Experiment 3:** THC & cannabinoid exposure (CME) (5, 15, 50 mg/kg/day, p.o.) or Sesame oil | Albino Rats | **Experiment 1:** GD14-P21  **Experiment 2:** GD15-P21  **Experiment 3:** GD6-15 | - **Experiment 1:** Tolerance observed after 3-5 days with initial & transient decrease in maternal weight gain (5 mg/kg). - No effect on gestational length, fetal mortality, fertility, litter size, fetal weight, or sex ratio at weaning. - **Experiment 2:** Increased mortality at 5 mg/kg. - Dose-independent differences in weight & sex ratios at weaning. - **Experiment 3:** THC & CME: Decreased maternal weight gain. - No fetal abnormalities. | 1. Wright PL, Smith SH, Keplinger ML, Calandra JC, Braude MC, 1976 |
| Cannabinoid exposure (Cannabis Extract, CE); 10 or 150 mg/kg/day, gastric intubation, g.i. or Olive oil  Positive control: prenatal alcohol (6 g/kg/day) exposure for comparison of birth weight data | Long Evans Rats | GD3-Birth/P21 & WEEK 11 | - Decreased food & water intact & maternal weight gain - Decreased pup weight at birth by ~ 10% at 150 mg/kg dose versus pair-fed controls - No effect on litter size & pup mortality at birth - Increased postnatal mortality & decreased neonatal weight on day 21. - Only female neonatal weight at day 21 (weaning) was < that of pair-fed controls by week 11 | 1. Abel EL, Dintcheff BA, Day N, 1980 |
| Cannabinoid exposure (Cannabichromene, CBC) 50 mg/kg/day, p.o. &/or THC (50 mg/kg/day, p.o.) or CBC 5% Olive Oil & 2.5% Acacia in Distilled Water | ICR Swiss mice | GD20-P5/P90 | - Decreased survival to weaning age & increased perinatal mortality in THC-exposed pinkies. - Decreased survival rate, associated with an increased incidence of cannibalism, in THC-treated dams. - Decreased level of mounting, intromission & ejaculation - Decreased mean seminal vesicle weight & seminal vesicle weight ratio | 1. Hatoum NS, Davis WM, Elsohly MA, Turner CE, 1981 |
| Cannabinoid exposure (Hashish Extract, HE); 40% THC, 45% cannabidiol (CBD), 20 mg/kg, 3x/week, p.o. or Olive oil | C3H/HeJ Mice | **Experiment 1:** GD0-birth (Mating-Birth; Multiple acute administration)  **Experiment 2:** P21-birth (12 weeks from weaning-mating; Chronic administration)  **Experiment 3:** 3 weeks after weaning only (Early administration) | - **Experiment 1 & 2:** Generalized sedation with acute HE-exposure & decreased sexual behavior & social explorations despite greater non-social activities with chronic HE-exposure. - Increased mean gestational length by day 1. - No effect on birth weight - Some pinkies died of unknown causes or cannibalization. - **Experiment 3:** Early HE had no effect on mating behavior. - **Experiments 1-3:** Dams conceived overnight. - Decreased nesting during pregnancy after multiple acute (Expt. 1) & chronic (Expt. 2) HE - Decreased nesting material transport in early HE-exposed pinkies (Expt. 3). - Increased or normal non-social activities - Delayed birth by 1 day after multiple acute & chronic HE - No effect on birth weight, but decreased litter size after chronic HE - No effects on birth weight or litter size with acute HE | 1. Frischknecht HR, Sieber B, Waser PG, 1982 |
| THC (20 mg/kg/day, p.o.) or Sesame oil | Sprague-Dawley Rats | 2 weeks Preconception-Lactation/P10, 20, 40 or 60 | - Decreased weight gain in THC-treated dams during the 1st & 3rd weeks of pregnancy; no effect on average litter size or the average number of stillborn pups. - Decreased live pups/litter at birth, average birth weight & body weight at day 10 in THC-exposed pups. - No effect on body weight at subsequent ages - Decreased brain weight in THC-exposed pups at days 10 & 60 | 1. Walters DE, Carr LA, 1986 |
| THC (25 or 50 mg/kg/day, s.c.) or Sesame oil | Swiss-Webster Mice | GD6-Birth/~ P21 | - Decreased litter size & dose-dependent decrease in birth weight - No effect on maternal weight gain - No effect on resorption rate | 1. Abel EL, Tan SE, Subramanian M, 1987 |
| THC (15 or 50 mg/kg/day, p.o.) or Sesame oil  *Pair-fed controls received only vehicle alone & ate & drank the same amount consumed by the 50 mg/kg group on the same gestation days, suggesting that decreased pup nipple attachment was not due to THC but was secondary to decreased food & water intake of dams.* | Wistar Rats | GD8-22/P32 | - No effect on locomotor activity - Increased latency to nipple attachment in pups from 50 mg/kg exposed dams & pair-fed controls on days 2, 5, 8, 11 & 14 | 1. Brake SC, Hutchings DE, Morgan B, Lasalle E, Shi T, 1987 |
| THC (15 or 50 mg/kg/day, g.i.) or Sesame oil | Wistar Rats | GD2-22/P57-60 | - Food & water intake was initially decreased to 75-80% in the 50 mg/kg group but then recovered to a 15-20% decrease over days 3-4 until term. - Decreased body weight from conception-term in THC & pair-fed control dams - Increased neonatal mortality in THC-exposed pups. - Dose-dependent increase in the male to female sex ratio & significant effects on rate of growth for both sexes | 1. Hutchings DE, Morgan B, Brake SC, Shi T, Lasalle E, 1987 |
| THC (15 or 30 mg/kg/day, p.o.) or Sesame oil | Wistar Rats | GD2-22/P57-60 | - No effect in response amplitude among males or females across groups - No effect in the rate of habituation of the response across groups over blocks of trials - No effect of dose or treatment on startle response amplitude was demonstrated | 1. Hutchings DE, Brake SC, Banks AN, Nero TJ, Dick LS, Zmitrovich AC, 1991 |
| THC (15 or 30 mg/kg/day, p.o..) or Sesame oil | Wistar Rats | GD2-22/P57-60 | - Decreased initial food & water intake (1^st^ two 30 mg/kg doses). - THC & pair fed groups: Decreased weight gain versus ad libitum & naïve controls. - No effect on implantation sites, resorptions, perinatal mortality, litter size, or sex ratio. - Positive correlation between increased total mortality (resorptions & perinatal mortality) & pair fed & THC groups. - Decreased male birth weight in pair fed & THC groups versus controls. - Decreased female birth weight in THC versus controls. | 1. Hutchings DE, Fico TA, Banks AN, Dick LS, Brake SC, 1991 |
| Acute Exposure: THC (5 mg/kg, p.o.) or Sesame oil  Chronic prenatal Exposure: THC (5 mg/kg/day, p.o.) or Sesame oil | Wistar Rats | GD13-P7/P7 | - Increased 5-HT content in dorsal hippocampus, substantia nigra & neostriatum; no effect on 5-HT content in cingulate cortex, raphe nuclei, locus coeruleus & anterior hypothalamus. - Decreased endogenous 5-hydroxyindole-3-acetic acid (5-HIAA) in anterior hypothalamus & raphe nuclei. - Increased 5-HT levels in the neostriatum & decreased 5-HT levels in anterior hypothalamus, raphe nuclei & locus coeruleus in acute THC-exposed pups. - Increased tissue 5-HIAA in anterior hypothalamus & substantia nigra - No effect on 5-HT uptake site density, as determined by [3H] paroxetine binding & increased 5-HT uptake in the cingulate cortex of perinatal-treated pups versus acutely treated pups. | 1. Molina-Holgado F, Molina-Holgado E, Leret ML, Gonzalez MI, Reader TA, 1993 |
| Cannabinoid exposure (HE) THC 20 mg/kg/day, p.o. or Sesame oil | Wistar Rats | GD5-P24/ P15, 20, 30, 40, 70 | - Increased mesencephalon TH-mRNA levels in HE-exposed males at days 15 & 20 & increased TH protein in mesencephalon but not striatum - No effect on mesencephalon TH-mRNA or TH protein levels at days 30 & 40 - Decreased TH-mRNA but not TH protein in mesencephalon or striatum in HE-exposed adult males | 1. Bonnin A, de Miguel R, Rodriguez-Manzaneque JC, Fernandez-Ruiz JJ, Santos A, Ramos JA, 1994 |
| THC (5 mg/kg/day, p.o.) or Sesame oil | Wistar Rats | GD5-P24/P15, 20, 30, 40, 70 | - Development of motor behaviors over time, sexually dimorphic alterations on specific days - Stress-induced motor behaviors altered in females. - Locomotor Activity: Increased rearing & time spent grooming on P15 & 20 in adult males & females; these behaviors normalized postweaning & restarted it P70 in females. - Decreased sociosexual approach in females - Increased dark-light emergence latency in females | 1. Navarro M, Rubio P, Rodriguez de Fonseca F, 1994 |
| THC (5 mg/kg/day, p.o.) or Sesame oil | Wistar Rats | GD5-P24/P15, 20 & 70 | - Increased locomotor activity & increased exploratory behavior at pre-weaning in males & females - Alterations in the behavioral response to novelty, social interactions, sexual orientation & sexual behavior in adult females. - No effect on habituation & reactivity to different illumination conditions. - Increased spontaneous & water-induced grooming behavior in adult male & female offspring. - Low dose THC increased morphine reward sensitivity in adult male & female offspring in a morphine conditioned place preference (MPP) paradigm. | 1. Navarro M, Rubio P, de Fonseca FR, 1995 |
| THC (5 mg/kg/day, p.o.) or Sesame oil  Morphine (350 µg/kg) or Saline Challenge | Wistar Rats | GD5-P24/P70 | - Increased morphine reward sensitivity in adult males & females - Increased open field behavior. - Increased locomotor activity in females - Increased exploratory behavior of males in a plus-maze paradigm | 1. Rubio P, Rodríguez de Fonseca F, Muñoz RM, Ariznavarreta C, Martín-Calderón JL, Navarro M, 1995 |
| THC (5 mg/kg/day, p.o.) or Sesame oil  P24: Naloxone (NAL; 5 mg/kg, i.p.) Challenge on P24 or Saline  P75: Morphine (MOR; 5 mg/kg, i.p.) Challenge on P75 or Saline | Wistar Rats | GD5-P24/P24, 50 &/or 75 | - Increased typical opioid-like withdrawal signs (palpebral ptosis, chewing, piloerection & wet-dog shakes) after NAL challenge in males. - Decreased basal sensitivity to radiant heat in neonatal (P24) & juvenile (P50) males. - Increased sensitivity to pain after MOR challenge in same adult males (P75 | 1. Vela G, Fuentes JA, Bonnin A, Fernández-Ruiz J, Ruiz-Gayo M, 1995 |
| THC (5 mg/kg/day, p.o.) or Sesame oil | Wistar Rats | GD5-P1/~ P21 | - Decreased diencephalic 5-HT levels in males versus females | 1. Molina-Holgado F, Amaro A, Gonzalez MI, Alvarez FJ, Leret ML, 1996 |
| Cannabinoid exposure (HE) 20 mg/kg/day, p.o.or Sesame oil | Wistar Rats | GD5-P24/Adult | - Increased time spent with incentive female & decreased time spent with incentive male (opposite of control males) - Increased time spent in active social interaction after high light level (neophobic) exposure in HE-exposed males. - No effect in the dark-light emergence test - Decreased limbic forebrain l-3,4-dihydroxyphenylacetic acid (DOPAC) content in adult males, which was correlated with the latency of 1^st^ visit | 1. Navarro M, de Miguel R, Rodríguez de Fonseca F, Ramos JA, Fernández-Ruiz JJ, 1996 |
| THC (5 mg/kg per day) | Wistar Rats | GD5-P24/P70 | - Decreased 5-HT levels in hypothalamus & rostral neostriatum in THC-exposed males. - Decreased 5-HT levels of in ventral hippocampus, septum & midbrain raphe nuclei in THC-exposed males & females. - Increased 5-HIAA levels in dorsal hippocampus, hypothalamus, septum, midbrain raphe nuclei & rostral neostriatum in THC-exposed males & females | 1. Molina-Holgado F, Alvarez FJ, Gonzalez I, Antonio MT, Leret ML, 1997 |
| THC (1 or 5 mg/kg/day, p.o.) or Sesame oil  Morphine (350 µg/kg) or Saline Challenge | Wistar Rats | GD5-P24/Adult | - Increased morphine (350 µg/kg) reward sensitivity in MPP paradigm in adult males (maternal THC 1 & 5 mg/kg) & females (maternal THC 1 mg/kg) - Increased male exploratory behavior defensive withdrawal. - Decreased anxiety-like behaviors in males - Increased basal corticosterone levels & decreased adrenal response to the HPA-activating in MPP paradigm in females (maternal THC 1 or 5 mg/kg) - Normal to decreased basal corticosterone levels & increased adrenal response in MPP paradigm in males (maternal THC 1 or 5 mg/kg) | 1. Rubio P, Rodríguez de Fonseca F, Martín-Calderón JL, Del Arco I, Bartolomé S, Villanúa MA, Navarro M, 1998 |
| THC (5 mg/kg/day, p.o.) or Sesame oil | Wistar Rats | GD5-P24/Adult | - No effect on food-reinforced behavior in adult males & females - Increased acquisition rate in morphine self-administration, fixed-ratio schedule in adult females - Decreased density of mu-opioid receptors (mOR) in the caudate-putamen & amygdala posteromedial cortical nucleus (PMCo) in males. - Increased density of mOR in the prefrontal cortex (PFC), hippocampal CA3 area, PMCo & ventral tegmental area (VTA), the periaqueductal grey matter in females & decreased binding in the lateral amygdala | 1. Vela G, Martin S, Garcia-Gil L, Crespo JA, Ruiz-Gayo M, Fernandez-Ruiz JJ, Garcia-Lecumberri C, Pelaprat D, Fuentes JA, Ramos JA, Ambrosio E, 1998 |
| THC (5 mg/kg/day, p.o.) or Sesame oil | Wistar Rats | GD5-P24/P70 | - Increased patterns of food & morphine self-administration (SA) in THC- & oil-exposed females versus males, corresponding to increased [dopaminergic](https://www.sciencedirect.com/topics/pharmacology-toxicology-and-pharmaceutical-science/dopamine-receptor-stimulating-agent) activity (DOPAC/DA) in the nucleus accumbens (NAC) before morphine SA compared to males. - Decreased DOPAC/DA in THC-exposed females versus oil-exposed females & similar to oil- & THC-exposed males - Decreased DOPAC/DA in the ventral tegmental area in THC-exposed females - All these changes normalized after 15 days of morphine SA & they did not reappear after 15 additional days of extinction of this response. | 1. González B, de Miguel R, Martín S, Pérez-Rosado A, Romero J, García-Lecumberri C, Fernández-Ruiz J, Ramos JA, Ambrosio E, 2003 |
| WIN55,212–2 (WIN; 0.5 mg/kg/day, s.c.) or 0.3% Tween 80 in Saline | Wistar Rats | GD5-20/P12-40 & 80 | - Decreased memory retention, in 40- & 80-day-old males subjected to a passive avoidance task, was correlated with decreased hippocampal CA3-CA1 long-term potentiation (LTP) & glutamate release. - Increased locomotor activity in males at 12 & 40 days - Decreased basal & K^+^-evoked extracellular hippocampal glutamate levels in WIN-exposed juveniles & adults born from treated dams. - Decreased glutamate outflow in primary hippocampal cell cultures from WIN-exposed pups | 1. Mereu G, Fà M, Ferraro L, Cagiano R, Antonelli T, Tattoli M, Ghiglieri V, Tanganelli S, Gessa GL, Cuomo V, 2003 |
| WIN (0.5 mg/kg, s.c.) or 0.3% Tween 80 in Saline  N-methyl-D-aspartic acid (NMDA; 0.01-10 um, 10 min) Challenge | Wistar Rats | GD5-20/P1, 10, 12, 80 | - No effect of WIN-exposure on dam weight gain, pregnancy length & litter size at birth - No effect on body weight & postnatal mortality of WIN-exposed male pups - No effect of NMDA Challenge on extracellular glutamate levels in cortical cell cultures from WIN-exposed pups at day 1 - Increased latency to reach the goal area in WIN-exposed pups on days 10 & 12. - Decreased rate of calling (i.e., # of USVs/5s) in day 10 pups removed from nest. - Decreased the acquisition of an active avoidance task (i.e., % of day 80 WIN-exposed rats achieving the criterion of 80% of conditioned avoidance responses (CARs) for 3 consecutive sessions) - Decreased growth pattern (i.e., minimal neuron population) in the cortical cultures from WIN-exposed pups - Neurite staining with MAP2 antibody revealed abnormal neurite outgrowth: decreased neurite branching & thinner & shorter neurites; altered MAP2 immunoreactivity (ir) distribution along neurites with areas of condensed MAP2 ir in some neurite areas & absent in other areas | 1. Antonelli T, Tomasini MC, Tattoli M, Cassano T, Tanganelli S, Finetti S, Mazzoni E, Trabace L, Steardo L, Cuomo V, Ferraro L, 2005 |
| WIN (0.5-1 mg/kg/day, s.c.) or 1% Tween 80 in Saline  P40 & 80: Apomorphine (APO; 0.25mg/kg, s.c.) Challenge or D[izocilpine](https://www.sciencedirect.com/topics/neuroscience/dizocilpine) (0.05, 0.1 mg/kg, s.c.) or Saline | Sprague-Dawley Rats | GD5-20/P 40, 60 & 80 | - No effect on prepulse inhibition (PPI) of the startle magnitude & sensorimotor gating in WIN-exposed pups at day 40, 60 or 80 - No effect on prepulse inhibition-disrupting effects of [apomorphine](https://www.sciencedirect.com/topics/neuroscience/apomorphine) & [dizocilpine](https://www.sciencedirect.com/topics/neuroscience/dizocilpine), suggesting that WIN prenatal exposure is likely unable to affect sensitivity of sensorimotor gating substrates to [dopaminergic](https://www.sciencedirect.com/topics/neuroscience/dopaminergic) agonists & [NMDA receptor antagonists](https://www.sciencedirect.com/topics/neuroscience/nmda-receptor-antagonist). | 1. Bortolato M, Frau R, Orrù M, Casti A, Aru GN, Fà M, Manunta M, Usai A, Mereu G, Gessa GL, 2006 |
| THC (5 mg/kg/day, p.o. via cannula) or Sesame oil | Wistar Rats | GD15-P9/P80 | - Decreased long-term memory in inhibitory avoidance test. - Decreased olfactory short-term memory in males in social discrimination test | 1. Campolongo P, Trezza V, Cassano T, Gaetani S, Morgese MG, Ubaldi M, Soverchia L, Antonelli T, Ferraro L, Massi M, Ciccocioppo R, Cuomo V, 2007 |
| THC (5 mg/kg/day, p.o.) or Sesame oil & Alcohol (EtOH; 3%/day)  Groups: EtOH, THC, or EtOH+THC  SR-141716A (0.3-3.0 mg/kg, i.p.) Challenge | Rats | GD15-P9/Adult | - No effect on alcohol self-administration & alcohol seeking in EtOH, THC, or EtOH+THC groups. - Decreased lever pressing for ethanol & blocked conditioned reinstatement of alcohol seeking after SR-141716A (0.3–3.0 mg/kg) - No effect of SR-141716A on foot-shock stress-induced reinstatement of alcohol seeking. | 1. Economidou D, Mattioli L, Ubaldi M, Lourdusamy A, Soverchia L, Hardiman G, Campolongo P, Cuomo V, Ciccocioppo R, 2007 |
| THC (0.15 mg/kg/day, i.v.) or 0.3% Tween 80 in Saline  Heroin (HER) Infusion (15g/kg/infusion & 30g/kg/infusion under a fixed-ratio 1 schedule of reinforcement  P62: HER (2 ml/kg, s.c.) Challenge or Saline  P83: SR 141716A (Rimonabant; 2 ml/kg i.p.) Challenge or 0.3% Tween 80 in Saline | Long Evans Rats | GD5-P2/P62 & 83 | - No effect on the % weight gain by the pregnant dams, gestational length, pup length at P2 & pup weight - No effect on weight between THC- or vehicle-treated with HER self-administration (SA) on P62 - No effect on HER SA with 15 g/kg/infusion & increase HER SA with 30g/kg/infusion under a fixed-ratio 1 schedule of reinforcement in THC-exposed adults (P62) - Increased active lever presses percentage after mild stress (1 day without food) in THC-exposed adults. - Decreased HER-induced locomotor activation & no effect on HER self-administration during normal conditions - Increased HER seeking in males during mild stress & extinction | 1. Spano MS, Ellgren M, Wang X, Hurd YL, 2007 |
| THC (2 mg/kg/day, s.c.) or Ethanol, Tween 80 & 0.9% Saline (1:1:18) | Long Evans Rats | GD1-22 & P2-10/P90 | - No effect on maternal weight gain, gestational length, or weight on P2 - No effect on locomotor activity - Increased anxiety-like behavior - Increased social interaction in males. - No effect on depression-like behavior | 1. Newsom RJ, Kelly SJ, 2008 |
| THC (2.5-5 mg/kg/day, p.o.) or Sesame oil via buccopharyngeal cannula | Wistar Rats | GD15-P9/P12, 35 & 80 | - No effect on maternal weight gain, gestational length, litter size, pup weight gain, or postnatal mortality. - No effect on locomotor activity - Increased isolation-induced ultrasonic vocalizations (USVs; 5 mg/kg) on P12 - Decreased social interaction & play behavior on P35. - Increased anxiety-like behavior of males in elevated plus-maze paradigm on P80 | 1. Trezza V, Campolongo P, Cassano T, Macheda T, Dipasquale P, Carratù MR, Gaetani S, Cuomo V, 2008 |
| THC (0.15 mg/kg/day, i.v.) or 0.3 % Tween 80 in Saline | Long Evans Rats | GD5-P2/P62 (Adult) | - Decreased dopamine receptor D2 (DRD2) mRNA expression in the NAC of THC-exposed adults - Increased the repressive dimethylated lysine 9 (2meH3K9) mark & upstream of the transcription start site (TSS) & decreased trimethylated lysine 4 (3meH3K4) on histone H3 across the genomic fragment in the NAC of THC-exposed adults. - Decreased RNA polymerase II (Pol II) at the TSS & within the coding region - No effect on 2meH3K9 at the DRD1 gene & decreased 3meH3K4 & decreased Pol II | 1. DiNieri JA, Wang X, Szutorisz H, Spano SM, Kaur J, Casaccia P, Dow-Edwards D, Hurd YL, 2011 |
| THC (0.15 mg/kg/day, i.v.) or Pluronic Acid in Saline    P60: Amphetamine (1mg/kg, i.p.) Challenge or Saline | Sprague Dawley Rats | GD1-21/P22, 45 & 60 | - Decreased retention in passive avoidance test on P22 - Decreased reversal in active avoidance test in males & no effect on attention on P45 - Decreased response in amphetamine challenge on P60 | 1. Silva L, Zhao N, Popp S, Dow-Edwards D, 2012 |
| THC (3 mg/kg/day, i.p.) or 3% DMSO & 2% Tween 80 in Saline | CB1R KO Mice | GD12.5-16.5/ P 2.5 & 20 | - Decreased subcerebral projection neuron development & corticospinal motor function & transiently down-regulated CB1Rs - Increased glutamatergic CB1R signal in pyramidal neuron fibers at P2.5 & decreased immunopositive puncta corresponding to glutamatergic (VGLUT1-positive) terminals at P20. - Increased CB1R signal corresponding to GABAergic (VGAT-positive) neurons in the hippocampal formation at P2.5 & mature cortex at P20. | 1. de Salas-Quiroga A, Díaz-Alonso J, García-Rincón D, Remmers F, Vega D, Gómez-Cañas M, Lutz B, Guzmán M, Galve-Roperh I, 2015 |
| Cannabinoid exposure (Marijuana Smoke, MS), 0.2 g of Cannabis, containing 0.3% delta-9-THC p.i./day or Filtered Air | Balb/C Mice | GD5.5-17.5/GD5.5-12.5 & GD13-18.5 (Maternal weight & food consumption measured)  GD10.5 & 16.5 (day dpc, Ultrasound Biomicroscopy) | - No effect on maternal weight gain, implantation, litter size, fetal growth, or fetal mortality - Decreased birth weight & cannabis-induced increased number of males versus females. - Decreased fetal-to-placental weight ratio in males. - Decrease in fetal lung, brain, thymus & liver | 1. Benevenuto SG, Domenico MD, Martins MA, Costa NS, de Souza AR, Costa JL, Tavares MF, Dolhnikoff M, Veras MM, 2017 |
| THC (5 mg/kg/day, i.p.) or 5% Ethanol & 5% Corn oil in Saline or WIN (0.75 mg/kg/day, i.p.) | WT C57/BL6 or Serotonin Reporter Mice (5-HT3AR-GFP or Nkx2.1-cre: RCE-GFP reporter lines) | GD10-18/Adult | - Decreased cholecystokinin (CCK)-INT density & altered CCK-interneuron (INT) morphology in THC or WIN-treated pups; no effect on parvalbumin, somatostatin, calretinin, or vasoactive intestinal peptide expressing INT densities & no effect on CAI pyramidal cell morphology or physiology. - Decreased CCK-INT mediated depolarization-induced suppression of inhibition (DSI) sensitive spontaneous inhibition, feedforward inhibition & feedforward inhibition in WIN-exposed pinkies at P14-25 & P30-45 - Decreased constitutive CB1R activity; no effect on other synaptic properties of residual CCK INTs - Decreased social interaction in three-chamber social interaction test in WIN-exposed pinkies | 1. Vargish GA, Pelkey KA, Yuan X, Chittajallu R, Collins D, Fang C, McBain CJ, 2017 |
| THC (5 mg/kg/day, s.c.) or 5% Ethanol, 5% Cremophor in Saline  WIN (0.5mg/kg, s.c.) or 5% DMSO & 5% Cremophor in Saline | Wistar Rats | GD5-20/Adult | - Decreased social interaction in males. - No effect on anxiety-like behavior - No effect on cognition | 1. Bara A, Manduca A, Bernabeu A, Borsoi M, Serviado M, Lassalle O, Murphy M, Wager-Miller J, Mackie K, Pelissier-Alicot AL, Trezza V, Manzoni OJ, 2018 |
| **Prenatal Methylazoxymethanol acetate (MAM)** **Exposure Model:**  MAM 22 mg/kg; i.p.) or Saline at GD17  **Perinatal THC Exposure Model:**  THC (5 mg/kg/day, p.o.) or Sesame oil at GD15-P9 | Sprague Dawley Rats | **MAM Model:** GD17/P180  **THC Model:** GD15-P9/P180 | - Increased behavioral deficits consistent with schizophrenia-like phenotype. - Decreased time spent in social behaviors in social interaction test (SIT) & no change in number of interactions with partner in MAM- & THC exposed pups - Increased cognitive deficits (i.e., decreased discrimination index) in novel object recognition test (NORT) in MAM- & THC-exposed pups - Increased lateral ventricles, increased blood perfusion in the circle of Willis (CoW) & sensorimotor cortex & decreased hippocampal perfusion in MAM-exposed pups. - No effect in all brain regions of interest (ROIs) in MAM- & THC-exposed pups - Association between perfusion of CoW & hippocampus in THC-exposed rats | 1. Drazanova E, Ruda-Kucerova J, Kratka L, Stark T, Kuchar M, Maryska M, Drago F, Starcuk Z Jr, Micale V, 2019 |
| THC (2 mg/kg/day, s.c.) or 1-2%Tween 80 in Saline | Sprague Dawley Rats | GD5-20/P15-28 | - Decreased sensorimotor gating in males but not females. - Increased pacemaker & evoked activity of VTA DA neurons in males - Decreased synaptic inhibition onto DA neurons in males. - Decreased number &/or strength of excitatory inputs terminating on DA neurons & increased amplitude of miniature excitatory postsynaptic currents & longer decay kinetics of postsynaptic AMPA currents. - Increased postsynaptic DA neuron responsiveness to excitatory stimuli in males. - Pregnenolone normalizes synaptic plasticity, deficits in DA neuron activity & restores behavior in males | 1. Frau R, Miczán V, Traccis F, Aroni S, Pongor CI, Saba P, Serra V, Sagheddu C, Fanni S, Congiu M, Devoto P, Cheer JF, Katona I, Melis M, 2019 |
| THC (2 mg/kg/day, s.c.) or 1-2% Tween 80 in Saline  P30-62: Alcohol (96% in tap water at 10% v/v) Challenge | Wistar Rats | GD5-20/P25 & 62 | - Increased locomotor activity & no effect on exploration or emotional reactivity in adolescent THC-exposed pups - No effect on response to natural reinforcing stimuli - Decreased spatial memory & object recognition memory adolescents in a reinforce-motivated can test. - Decreased spatial memory & reversal learning in an aversive context in Barnes maze test. - Increased hippocampal expression levels of N-methyl-D-aspartate receptor (NMDAR) NR1 subunit & decreased NR2A & postsynaptic density protein 95 in the adolescent males. - Increased hippocampal metabotropic glutamate receptor 5 (mGluR5) & its scaffolding partner, Homer protein homolog 1 (HOMER1) protein isoform in adolescents. - Increased hippocampal CB1R & HINT1 in adolescent males | 1. Brancato A, Castelli V, Lavanco G, Marino RAM, Cannizzaro C, 2020 |
| Vaporized Ethanol (EtOH; 95%, 10 L/min airflow) or Air for 3 h  EtOH-exposed dams were exposed to THC (100 mg/mL at 2 L/min airflow) or Propylene glycol (VEH) for 6-s puff every 5 min for 30 min (7 puffs total) via an e-cigarette tank.  Groups: EtOH+THC; EtOH+VEH; Air+THC; Air+VEH | Sprague-Dawley Rats | GD5-20/GD5, 10, 15 20 (Blood Collection)  P30 (Body Weight Measurement) | - Increased blood alcohol concentrations in EtOH+THC-exposed pups - Increased THC levels over pregnancy & increased THC-OH metabolite levels versus THC-exposed pups on GD10 & 15 - No effect of THC exposure on gestational length, litter size, sex ratio, or birth weight. - Decreased body weights in THC-exposed adolescents | 1. Breit KR, Rodriguez CG, Lei A, Thomas JD, 2020 |
| THC (3 mg/kg/day, i.p.) or 3% DMSO & 2% Tween 80 in Saline | CB1 Conditional KO Mice | GD10.5-17.5/P60 | - Decreased dorsal hippocampal CB1R levels in THC-exposed wild-type pinkies. - Decreased CCK-containing interneurons, hippocampal oscillations, brain hyperexcitability & decreased spatial memory in males. - No effect on object recognition | 1. de Salas-Quiroga A, García-Rincón D, Gómez-Domínguez D, Valero M, Simón-Sánchez S, Paraíso-Luna J, Aguareles J, Pujadas M, Muguruza C, Callado LF, Lutz B, Guzmán M, de la Prida LM, Galve-Roperh I, 2020 |
| WIN (0.5 mg/kg/day, s.c.) or 5% Polyethylene Glycol, 5% Tween 80 & 90% Saline  CDPPB (3-cyano-N-(1-3-diphenyl-1H-pyrazol-5-yl) benzamide; .5 mg/kg, i.p.) or 5% Tween 80, 5% Polyethylene Glycol in Saline | Wistar Rats | GD5-20/P10 & 13 | - No effect on pregnancy length, litter size at birth, postnatal viability & pup weight gain at P10, 13, 25 or 45 - Decreased social communication & locomotion WIN-exposed in pups. - Decreased isolation induced USVs at P10 when separated from the dam & siblings in males. - Increased crossing test area frequency in homing behavior test in males - Increased locomotor activity in males who were spared after treatment with CDPPB, a positive allosteric modulator of the mGlu5 receptor (metabotropic glutamate receptor 5) - No effect on social interaction, anxiety-like behavior, or temporal order memory | 1. Manduca A, Servadio M, Melancia F, Schiavi S, Manzoni OJ, Trezza V, 2020 |
| THC (3 mg/kg/day, i.p.) or Cremophor: Saline (1:18) | Wistar Rats | GD6.5 to GD22/GD19.5 (Placenta Assessed)  GD22 (Pregnancy & Neonatal Outcomes Measured) | - No effect on gestational or length litter size. - Increased symmetrical fetal growth restriction (decreased weight & length) - Decreased birth weight & heart: body weight, liver: body weight & brain: body weight ratios - Decreased placental weight ratio, increased placental labyrinth layer, decreased labyrinth progenitor expression & increased labyrinth-specific vascular defects | 1. Natale BV, Gustin KN, Lee K, Holloway AC, Laviolette SR, Natale DRC, Hardy DB, 2020 |
| Cannabinoid exposure (Cannabis Vapor, CAN_THC_: 400 mg/ml/2x/day, p.i., Vehicle Vapor (VEH), or No Vapor (AIR) or 80% Propylene glycol/20% Vegetable glycerol via apparatus | Long-Evans Rats | Preconception up to P6/P26-Adult | - No effect on litter size - Decreased weight gain in CAN_THC_ & VEH pups prior to weaning. - Increased isolation induced USVs (i.e., increased emotional reactivity) on P6. - Decreased social investigation [behaviors](https://www.sciencedirect.com/topics/neuroscience/behavior-neuroscience) in males on P26 - Decreased time exploring open arms of [elevated plus maze](https://www.sciencedirect.com/topics/neuroscience/elevated-plus-maze) & dose-dependent decreased behavioral flexibility in attentional set-shifting task in adults | 1. Weimar HV, Wright HR, Warrick CR, Brown AM, Lugo JM, Freels TG, McLaughlin RJ, 2020 |
| THC (5 mg/kg/day, p.o.) or Sesame oil  P19-39: Cannabinoid exposure (CBD; 30/mg/kg/day, i.p.) or Sesame oil | Sprague-Dawley Rats | GD15-P9/P1-12 (Behavioral tests & neurochemical analyses)  GD15-P9/P 100 (Behavioral tests & neurochemical analyses) | - Decreased neonatal reflexes, time in maternal nest area & no effect on exploration in THC-exposed rats. - Decreased whole brain content of 2-arachidonoylglycerol (2-AG) & increased monoacylglycerol lipase (MGL) transcript levels at P10 - Increase fatty acid amide hydrolase (FAAH) mRNA expression & no effect on anandamide (AEA), N-palmitoylethanolamide (PEA), or N-oleoylethanolamide (OEA) brain levels. - Increased DRD2 mRNA expression with decreased DNA methylation of the DRD2 regulatory region in the 6 CpGs & increased D2 receptor expression in adults - Decreased DNA methylation but no effect on mRNA or protein expression in adults | 1. Di Bartolomeo M, Stark T, Maurel OM, Iannotti FA, Kuchar M, Ruda-Kucerova J, Piscitelli F, Laudani S, Pekarik V, Salomone S, Arosio B, Mechoulam R, Maccarrone M, Drago F, Wotjak CT, Di Marzo V, Vismara M, Dell'Osso B, D'Addario C, Micale V, 2021 |
| THC (3mg/kg/day, i.p.) or Cremophor: Saline (1:18) | Wistar Rats | GD6-22/P1 & 21 | - Decreased heart weight at birth with postnatal catch-up growth at 3weeks - 3-week echocardiogram: increased anterior left ventricular wall thickness during systole - Decreased stroke volume & cardiac output | 1. Lee K, Laviolette SR, Hardy DB, 2021 |
| THC (3 mg/kg/day, i.p.) or Cremophor: Saline (1:18) | Wistar Rats | GD6.5-22/P50-60 | - No effect on maternal food intake or weight gain or gestational length or litter size - Decreased liver weight: body weight ratio at birth in THC-exposed pups exhibited & liver weight normalized by week 3 - Increased visceral adiposity & hepatic dyslipidemia in THC-exposed adults. - Increased hepatic triglyceride levels with increased diglycerol acyltransferase & p66Shc, a member of the Src homologous-collagen homologue adaptor protein family, protein levels in the livers of male THC-exposed adults. - No effect on mitochondrial transcription factor A, phosphorylated pyruvate dehydrogenase to total PDH, lactate dehydrogenase subunit A & citrate synthase levels (i.e., proteins involved in aerobic metabolism) in THC-exposed adults at month 6. - Decreased protein levels of superoxide dismutase (SOD) 1 at month 6 & no effect on catalase, SOD2, 4-hydroxynonenol (4HNE), an indicator of lipid peroxidation, in THC-exposed males - No effect on catalase, SOD1 or SOD2 protein levels at week 3 in THC-exposed males & increased hepatic lipid peroxidation as indicated increased 4HNE levels. - Increased mitochondrial electron transport chain complexes at week &3 month 6 in THC-exposed males. - Decreased hepatic transcript levels of miR-203a-3p & miR-29a/b/c in THC-exposed adult males. | 1. Oke SL, Lee K, Papp R, Laviolette SR, Hardy DB, 2021 |
| THC (2 mg/kg/day, s.c.) or 1-2%Tween 80 in Saline | Sprague Dawley Rats | GD5-20/ P15-28 (Prepuberty) | - Increased firing frequency of putative VTA DA cell in males - Decreased population activity of tonically active VTA DA neurons - Increased sensitivity to DRD2 activation & vulnerability to acute stress due to decreased sensory motor gating function | 1. Sagheddu C, Traccis F, Serra V, Congiu M, Frau R, Cheer JF, Melis M, 2021 |
| THC (2 mg/kg/day, s.c.) or 1-2%Tween 20 in Saline | Sprague Dawley Rats | GD5-20/P28-40 | - No effects on locomotor activity in an open field arena at baseline or after acute THC, anhedonia, emotional memory, or risk taking, thigmotaxis (i.e., movement toward a solid object; indicator of anxiety-like), or social behaviors (i.e., time spent in active or passive activities, social interaction/exploration) or sucrose preference. - Increased accumbal DA levels in preadolescent females - No effect on basal extracellular DA levels or intrinsic or excitatory synaptic properties of putative VTA DA neurons in preadolescent females. | 1. Traccis F, Serra V, Sagheddu C, Congiu M, Saba P, Giua G, Devoto P, Frau R, Cheer JF, Melis M, 2021 |
| Cannabinoid exposure (CS) Cigarette (0.3% THC, p.i.) or Filtered Air | BALB/c Mice | GD5.5-17.5/P20 & 60 | - Increased polycyclic aromatic hydrocarbons characterized in CS gaseous & particulate samples. - Decreased olfactory bulb & diencephalon volume in male CS-exposed fetuses; increased brain volume in PND60 pinkies. - Decreased thalamus & hypothalamus volume in P60 pinkies & increased cerebellum - Increased cortical BDNF immunoreactivity in CS-exposed pinkies at P60 - Protein expression analysis: increased fetal brain pro-BDNF & increased mature BDNF in the PFC & increased PFC CB1R expression P60 - Increased hippocampal neuronal [nuclear](about:blank) [antigen](about:blank), NeuN, expression in CS-exposed adults - Decreased cell viability after cortical primary neurons exposed to CS | 1. Benevenuto SGM, Domenico MD, Yariwake VY, Dias CT, Mendes-da-Silva C, Alves NO, Caumo SEDS, Vasconcellos P, Morais DR, Cardoso MS, Ianicelli J, Waked D, Davey GP, Boylan F, Costa JL, Veras MM, 2022 |
| Nicotine (NIC; 36mg/ml, p.i.), THC (100mg/ml, p.i.), THC+NIC, or Propylene glycol via e-cigarettes | Sprague Dawley Rats | GD5-20/GD22-P2 | - Decreased plasma NIC & coNIC levels in NIC+THC-exposed pregnant dams than NIC-exposed dams - Decreased plasma THC & THC metabolites (THC-OH & THC-COOH) levels than THC-exposed dams - Decreased initial core body temperature with NIC-exposure & decreased temperature with THC-exposure. - No effects on maternal food or water intake, or weight gain - No effect of NIC, THC, or NIC+THC on basic litter outcomes: gestational length, number of pups born, sex ratio of litters, or average pup weight at birth - No effects in eye opening, a developmental milestone | 1. Breit KR, Rodriguez CG, Hussain S, Thomas KJ, Zeigler M, Gerasimidis I, Thomas JD, 2022 |
| Vaporized EtOH), THC via e-cigarettes, EtOH+THC, or Propylene glycol via e-cigarette for 30 min (airflow of 2 L/ min) | Sprague-Dawley Rats | GD5-GD20/P12-20 (Early Sensorimotor Development, ESD, Paradigm)  P30-32 (Parallel Bar Motor Coordination Paradigm)  P31-34 (Open-Field Activity Paradigm) | - Decreased weight throughout the ESD testing on P12-20 & parallel bar testing on P30-32, alone or with EtOH. - Decreased body weight of EtOH-exposed pups on P32 - Decreased weight in THC-exposed pups in open-field activity testing | 1. Breit KR, Rodriguez CG, Lei A, Hussain S, Thomas JD, 2022 |
| THC (0.15 mg/kg/day, i.v.) or 0.3 % Tween 80 in Saline | Long Evans Rats | GD5-P2/P62 | - Increased motivation for food in THC-exposed pups, increased learned helplessness, anhedonia & altered stress sensitivity. - Increased KMT2A (histone-lysine N-methyltransferase 2A) expression, which targets H3K4 (lysine 4 on histone H3) in cellular chromatin, in males | 1. Ellis RJ, Bara A, Vargas CA, Frick AL, Loh E, Landry J, Uzamere TO, Callens JE, Martin Q, Rajarajan P, Brennand K, Ramakrishnan A, Shen L, Szutorisz H, Hurd YL, 2022 |
| THC (100mg/ml/day, p.i.), Nicotine (NIC; 36mg/ml/day, p.i.), THC+NIC, or Propylene glycol for 40-min vapor inhalation session (2 L/min airflow; one 6s puff every 5 min for 30 min followed by a 10-min post-exposure air clearance | Sprague Dawley Rats | GD5-20/P12-20 & 30-32 | - Delayed sensorimotor development in THC+NIC-exposed pups. - No effect on sensorimotor development in THC-exposed pups - Decreased motor coordination in NIC- or THC-exposed pups, whereas NIC+THC exposure exacerbated these effects in females | 1. Hussain S, Breit KR, Thomas JD, 2022 |
| THC (5 mg/kg/day, oral gavage.) or Sesame oil  Nicotine vapes (5 mg/ml via e-cigarette) or Propylene glycol & Vegetable oil (50:50) | Wistar Rats | GD1-20/P1, 15 & 21, 35 & 37 | - Decreased body weight at birth in nicotine vape- or oral THC-exposed pups. - Decreased baseline startle reactivity in nicotine vape-exposed adolescent males &females & enhanced sensorimotor gating in the prepulse inhibition test in females. - Decreased prepulse inhibition in nicotine & THC co-exposed males - Decreased short-term memory in males prenatally exposed to THC with or without nicotine co-exposure & in THC-exposed females. - Increased anxiety-associated behaviors in THC- or nicotine-exposed males in the latency to approach a novel palatable food | 1. Lallai V, Manca L, Sherafat Y, Fowler CD, 2022 |
| THC (2mg/kg/day, s.c.) or 1-2%Tween 80 in Saline | Wistar Rats | GD5-20/P35-46 | - Increased locomotor activity & no effect on exploration in adolescents - No effect on emotional reactivity or responsivity to natural reinforcing stimuli - Decreased Spatial Memory & object recognition memory in adolescents in reinforce-motivated can test. - Decreased spatial memory & reversal learning in an aversive context. - Increased relative expression of hippocampal NMDAR NR1 subunit, decreased expression of NRA2 subunit & PSD-95, increased expression of mGluR5, HOMER1 protein, CB1R & HINT1 | 1. Castelli V, Lavanco G, Feo S, D'Amico C, Micale V, Kuchar M, Plescia F, Brancato A, Cannizzaro C, 2023 |
| THC (5 mg/kg/day, i.p.) or 5% Tween-80 in Saline  *Neural stem cell (NSC) differentiation: Pretreatment with SR141716 (Rimanonbant, RIM; 2 um for 1 hour, followed by treatment for 24 h with THC (7.5 um)* | C57BL/6 Mice | GD5.5–12.5/P21 | - Decreased hippocampus neuron development: increased Glial fibrillary acidic protein (GFAP) expression & decreased NeuN expression in the THC-exposed pinkies - RNA-sequencing of differentially expressed hippocampal gene analysis: downregulated genes related to neuron differentiation regulation, neuron generation, axon development & cell morphogenesis; upregulated related to nervous system development, neurogenesis & positive regulation cellular processes. - Decreased NSC proliferation & cell necrosis at high doses. - Increased the differentiation of NSCs into glial cells. - Quantitative RT-PCR: increased expression of neuron-associated gene MAP2 & glial cell- associated gene GFAP; Immunofluorescence: decrease of MAP2 expression & increase of GFAP expression after knockdown of MEF2C like THC-exposed cells. - Quantitative RT-PCR: decreased CB1R mRNA after RIM, a CB1R antagonist, treatment; decreased GFAP expression & increased trend in MAP2 expression after THC exposure, RIM pretreatment. - Increased NESTIN, a stem cell-related gene & MEF2C mRNA expression normalized after RIM pretreatment | 1. Peng H, Li H, Wei Y, Zhang R, Chang X, Meng L, Wang K, He Q, Duan T, 2023 |
| THC (3 mg/kg/day, i.p.) or vehicle | Wistar Rats | GD7-22/P21 & P70-120 | - Male and female offspring display long term deficits in various cognitive domains, these phenotypes were associated with highly divergent, sex-dependent mechanisms - Electrophysiological recordings revealed hyperactive PFC pyramidal neuron activity in both males and females, but hypoactivity in the ventral hippocampus (vHIPP) in males and hyperactivity in females - Cortical oscillatory activity states of theta, alpha, delta, beta, and gamma bandwidths were strongly sex divergent - P120 disturbances in dopamine D1R/D2 receptors, NMDA receptor 2B, synaptophysin, gephyrin, GAD67, and PPARα selectively in the PFC and vHIPP, in both regions in males, but only the vHIPP in females - Using matrix-assisted laser desorption/ionization imaging mass spectrometry (MALDI IMS), identified region-, age-, and sex-specific deficiencies in specific neural PUFAs, namely docosahexaenoic acid (DHA) and arachidonic acid (ARA), and related metabolites, in the PFC and hippocampus (ventral/dorsal subiculum, and CA1 regions). | 1. Sarikahya MH, Cousineau SL, De Felice M, Szkudlarek HJ, Wong KKW, DeVuono MV, Lee K, Rodríguez-Ruiz M, Gummerson D, Proud E, Ng THJ, Hudson R, Jung T, Hardy DB, Yeung KK, Schmid S, Rushlow W, Laviolette SR, 2023. |

s.c., subcutaneous; i.p., intraperitoneal; p.o.; oral gavage per os; p.i., per inhalation; i.v., intravenously; g.i., gastric intubation; GD., gestational day; P., postnatal day
